# The acid-base-nucleophile catalytic triad in ABH-fold enzymes is coordinated by a set of structural elements

**DOI:** 10.1371/journal.pone.0229376

**Published:** 2020-02-21

**Authors:** Alexander Denesyuk, Polytimi S. Dimitriou, Mark S. Johnson, Toru Nakayama, Konstantin Denessiouk

**Affiliations:** 1 Structural Bioinformatics Laboratory, Biochemistry, Faculty of Science and Engineering, Åbo Akademi University, Turku, Finland; 2 Institute for Biological Instrumentation of the Russian Academy of Sciences, Federal Research Center “Pushchino Scientific Center for Biological Research of the Russian Academy of Sciences”, Pushchino, Russia; 3 Department of Biomolecular Engineering, Graduate School of Engineering, Tohoku University, Sendai, Miyagi, Japan; Weizmann Institute of Science, ISRAEL

## Abstract

The alpha/beta-Hydrolases (ABH) are a structural class of proteins that are found widespread in nature and includes enzymes that can catalyze various reactions in different substrates. The catalytic versatility of the ABH fold enzymes, which has been a valuable property in protein engineering applications, is based on a similar acid-base-nucleophile catalytic mechanism. In our research, we are concerned with the structure that surrounds the key units of the catalytic machinery, and we have previously found conserved structural organizations that coordinate the catalytic acid, the catalytic nucleophile and the residues of the oxyanion hole. Here, we explore the architecture that surrounds the catalytic histidine at the active sites of enzymes from 40 ABH fold families, where we have identified six conserved interactions that coordinate the catalytic histidine next to the catalytic acid and the catalytic nucleophile. Specifically, the catalytic nucleophile is coordinated next to the catalytic histidine by two weak hydrogen bonds, while the catalytic acid is directly involved in the coordination of the catalytic histidine through by two weak hydrogen bonds. The imidazole ring of the catalytic histidine is coordinated by a CH-π contact and a hydrophobic interaction. Moreover, the catalytic triad residues are connected with a residue that is located at the core of the active site of ABH fold, which is suggested to be the fourth member of a “structural catalytic tetrad”. Besides their role in the stability of the catalytic mechanism, the conserved elements of the catalytic site are actively involved in ligand binding and affect other properties of the catalytic activity, such as substrate specificity, enantioselectivity, pH optimum and thermostability of ABH fold enzymes. These properties are regularly targeted in protein engineering applications, and thus, the identified conserved structural elements can serve as potential modification sites in order to develop ABH fold enzymes with altered activities.

## Introduction

The structural family of alpha/beta-Hydrolases (ABH) includes enzymes that are widely found in nature and have diverse functions, such as esterases, lipases, thioesterases, amidases, epoxide hydrolases, dehalogenases, haloperoxidases, and hydroxynytrile lyases [1–3]. Because of their remarkable catalytic versatility, the ABH fold enzymes have often served as targets in protein engineering applications for the development of biocatalysts with improved characteristics [4]. Nevertheless, the ability of the ABH fold enzymes to catalyze various reactions in different substrates is based on a similar acid-base-nucleophile catalytic mechanism that is located at the core of the ABH fold [5–7].

**Fig 1:**
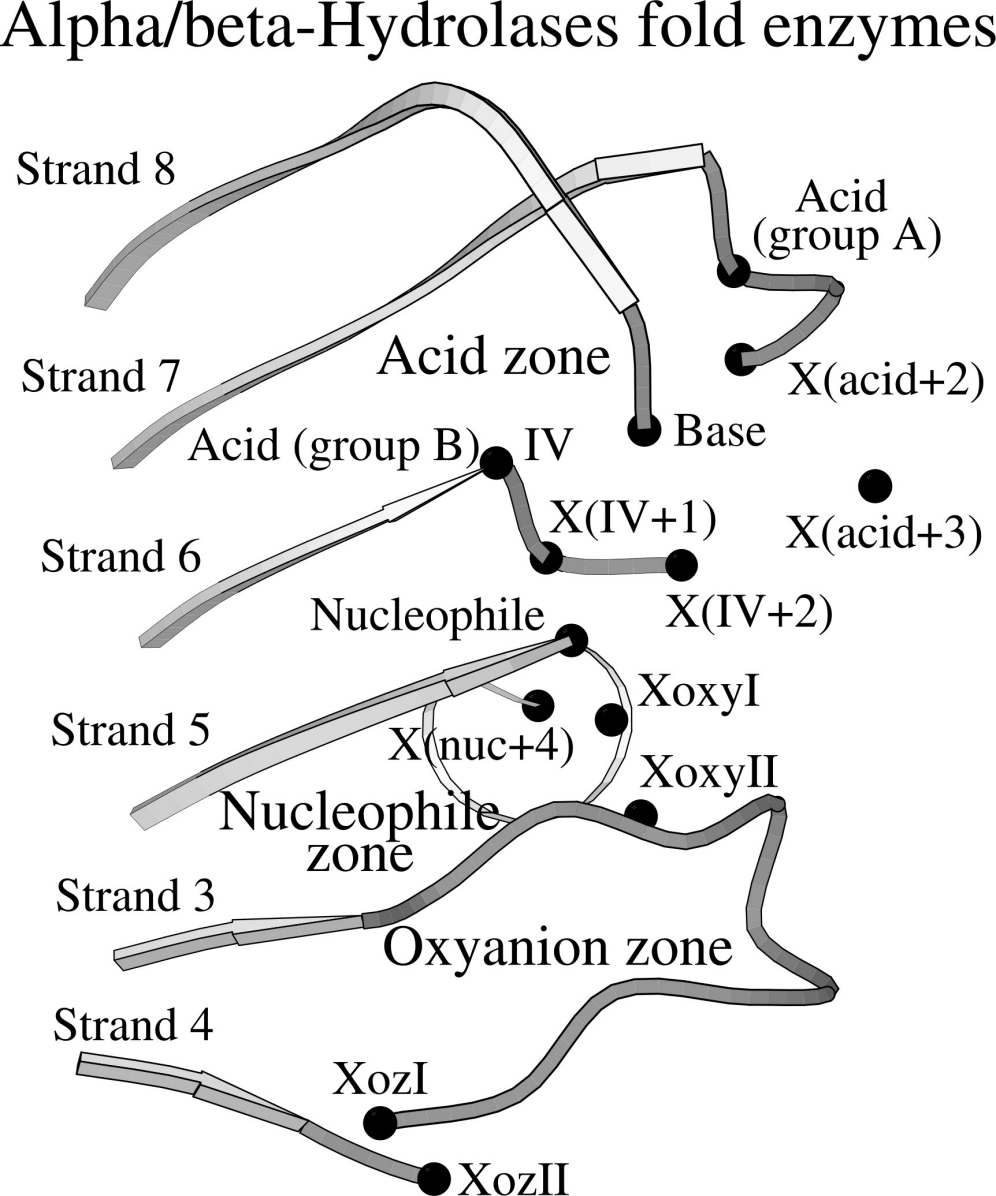
Conserved structural motifs at the catalytic site of ABH fold enzymes. Most key features of the catalytic machinery are coordinated by conserved structural organizations within the same plane as the central β-sheet of the ABH fold. Specifically, the catalytic acid (“Acid”), which is located at the turn that follows strand β7 (“group A”) or at the C-terminus of strand β6 (“group B”, position “IV”), is coordinated by the structural organization that is called “Catalytic acid zone” (“Acid zone”), while the catalytic nucleophile (“Nucleophile”) and the two residues that help form the oxyanion hole (“XoxyI” and “XoxyII”) are coordinated by the overlapping “Nucleophile zone” and “Oxyanion zone”. The catalytic histidine (“Base”) is located at a flexible loop that follows strand β8 and is linked with the β-sheet at the C-terminus of strand β6. The residues of the catalytic machinery, two conserved residues of Oxyanion zone (“XozI” and “XozII”), four residues of catalytic acid loops (“X(acid+2)”, “X(acid+3)”, “X(IV+1)” and “X(IV+2)”) and one residue of the nucleophile elbow (“X(nuc+4)”) are indicated with black dots.

The ABH fold is shaped by a β-sheet of eight mostly parallel β-strands, with the exception of the antiparallel strand β2, and is surrounded by six α-helices that are arranged in an α-turn-β supersecondary structure geometry, starting with αA helix that is situated before strand β4. Helical caps and other domains can be inserted in the fold, yet it is the ABH fold structure that comprises the catalytic domain of the ABH enzymes, with the residues of the acid-base-nucleophile catalytic mechanism being located at conserved positions across the ABH fold (Fig 1) [1, 6, 7]. Particularly, the catalytic acid is usually located at the turn after strand β7 (in the group A of the ABH fold enzymes) or alternatively, at the C-terminus of strand β6 (in the group B); the catalytic base, a conserved histidine, is located at a flexible loop after strand β8; and the catalytic nucleophile is located at the apex of a sharp turn at the C-terminus of strand β5, at a conserved structure called “nucleophile elbow” that retains structural and sequence conservation (G-X-Nuc-X-G motif). Located opposite to the catalytic triad, the oxyanion hole is usually shaped by two residues, one of which follows the catalytic nucleophile and the second is located at the C-terminus of strand β3 [1, 6, 7].

Based on the findings of our previous research, it appears that the invariable arrangement of the residues of the catalytic triad and the oxyanion hole on the ABH fold is not the only conserved structural feature of the catalytic mechanism of ABH fold enzymes. Indeed, in our research, where we are concerned with the structural conservation of the catalytic core of ABH fold enzymes, we have previously found that the residues involved in the catalytic machinery, which are located within the same plane of the β-sheet, not only maintain their structural positions on the ABH fold but are also surrounded by conserved, planar geometries [8, 9]. Specifically, “the catalytic acid zone” [8], “the nucleophile zone” [9] and “the oxyanion zone” [9] coordinate the catalytic acid, the catalytic nucleophile and the residues of the oxyanion hole respectively in the optimal arrangement that is required to achieve enzymatic activity. The planar zones are located at the C-termini of the strands of the β-sheet: the catalytic acid zone is formed by residues at the C-terminus of strand β7 and β6, while the nucleophile zone is formed by residues at the C-termini of strands β5 and β3 and overlaps with the oxyanion zone, which is formed by residues at the C-termini of strands β3 and β4.

In addition to the coordination of the catalytic acid, the catalytic nucleophile and the residues of the oxyanion hole, we have found that the catalytic acid zone, the nucleophile zone and the oxyanion zone indirectly affect the coordination of the catalytic histidine [8, 9]. In particular, we have shown that the catalytic acid zone is connected to the loop fragment prior to the catalytic histidine, and that conserved aromatic residues from the nucleophile zone and the oxyanion zone can interact with residues at the loop fragment that follows the catalytic histidine. We have deduced that the three planar zones have a cooperative effect on the stabilization of the flexible loop that hosts the catalytic histidine, regulating its position on top of the β-sheet and close to the catalytic acid and the catalytic nucleophile. The interactions that involve the loop fragments that precede and follow the catalytic histidine are complementary to the well-known, conserved weak-hydrogen bond that is observed in serine hydrolases [10], which anchors the imidazole ring of the catalytic histidine at the β-sheet by interacting with the main-chain oxygen of the residue at the C-terminus of strand β6.

So far, our comparative analysis of the structural core of ABH fold enzymes has been successful in identifying conserved, structural organizations that coordinate the units of the catalytic mechanism that are situated at the β-sheet. But, unlike the other residues of the catalytic mechanism, the catalytic histidine cannot be coordinated by a closed, structural organization because it is located at a flexible loop, and thus, a reasonable question is to ask whether there are structural elements that coordinate the catalytic histidine next to the other residues of the catalytic triad in addition to the direct and indirect interactions that we have mentioned above.

To address this question, we have focused here on the local structure around catalytic histidine, seeking for structural determinants that govern the fine tuning of the catalytic residues with respect to each other. The comparative analysis of the active sites of representative ABH structures has revealed several conserved structural elements that coordinate and properly orient the catalytic histidine next to the catalytic acid and the catalytic nucleophile. Even more so, it seems that several of the conserved structural elements that coordinate the residues of the catalytic mechanism, are routinely involved in ligand binding. Altogether, the results of this comprehensive study complete our structural description of the catalytic structural core of the ABH fold. Combined with information that arise from sequence comparisons and experimental data, we expect that the complete, structural mapping of the active site framework of ABH fold enzymes will help to shed light on the factors that affect the enzymatic activity, and potentially advance the utility of ABH fold enzymes in protein engineering applications.

## Results

As we have reviewed in the Introduction section, the residues of the acid-base-nucleophile catalytic triad of the ABH fold enzymes are located at conserved positions across the ABH fold: the catalytic acid and the catalytic nucleophile are located at the same plane with the β-sheet, while the catalytic base, a conserved histidine, is located outside this plane, but directly interacts with the other two catalytic residues. In our previous research, we have identified the conserved structural elements that coordinate the planar catalytic residues. Here, we have searched for conserved structural elements that coordinate the catalytic histidine, located next to both the catalytic acid and the catalytic nucleophile by comparing representative structures from the 40 ABH fold enzyme families of the Structural Classification of Proteins (SCOP) database [11], obtained from the Protein Data Bank (PDB) [12].

In order to explore the coordination of the catalytic histidine relative to the catalytic nucleophile and the catalytic acid, our comparative analysis has been conducted in two parts: the first part is concerned with the structure around the catalytic nucleophile-base pair, and the second part is concerned with the structure that surrounds the catalytic acid-base pair. The findings of this analysis are also presented in two parts: in the first part, we introduce the conserved structural elements that are involved in the coordination of the catalytic nucleophile next to the catalytic histidine, while in the second part, we revisit the area that surrounds the catalytic acid zone and the conserved structural elements that coordinate the catalytic acid next to the catalytic histidine. Additional conserved structural elements that are located at the active site of ABH enzymes, but are not involved in the coordination of the catalytic residues, are mentioned in both parts.

Lastly, we introduce our findings from the comparison of the active sites of ligand-bound ABH structures, where we have found that several of the conserved structural elements that are involved in the coordination of the catalytic residues also participate in the binding of the ligand.

### Coordination of the catalytic nucleophile next to the catalytic histidine

Our search for structural elements that support the contact between the catalytic nucleophile and the catalytic histidine is focused on the area where the two catalytic residues normally interact, i.e. around the C-termini of strands β5 and β6 (Fig 1). The C-terminus of strand β5 is part of the characteristic ABH-fold structural motif, known as “the nucleophile elbow”, with the catalytic nucleophile being located at the apex of the sharp turn that occurs at the C-terminus of strand β5. The C-terminus of strand β6 is situated parallel to the nucleophile elbow, followed by a similar sharp turn; the residue that is located at the apex of the sharp turn forms a weak hydrogen bond with the catalytic histidine, linking the imidazole ring with the β-sheet, as is often seen in serine hydrolases [10]; this same residue is a structural element of the catalytic acid zone, located at the position called “position IV” [8]. In most ABH fold families, the residue at position IV is in contact with the catalytic acid that is located at the turn after strand β7, but it is possible for the catalytic acid to alternately occur at position IV.

The comparison of the geometries near the edges of strands β5 and β6 in representative structures from 40 ABH fold enzyme families has revealed two conserved, weak hydrogen bonds that coordinate the catalytic nucleophile next to the catalytic histidine, through the connection of the catalytic nucleophile with positions IV and IV+1 located at the C-terminus of strand β6, which we refer to as “residue X_IV_” and “residue X_IV+1_” (Table 1).

**Table 1:** Inventory of interactions that participate in the proper positioning of the catalytic histidine next to the catalytic nucleophile in 40 ABH fold enzyme families. The main-chain oxygen atom of residue X_IV_ interacts with both the catalytic histidine and the catalytic nucleophile (column CA/Nucleophile–O/X_IV_), orienting the imidazole ring of the catalytic histidine with respect to the side-chain of the catalytic nucleophile. Residue X_IV+1_ also interacts with the catalytic nucleophile (column O/Nucleophile–CA/X_IV+1_) and thus, has an indirect influence on the optimal arrangement of the catalytic histidine-nucleophile pair. Unrelated to the coordination of the catalytic residues, an additional conserved contact (column Sc/(Nucleophile+4)–O/X_IV+2_) is formed between the residue that is located four positions after the catalytic nucleophile and the residue that is located two positions after residue X_IV_; this conserved contact is formed close to the catalytic nucleophile, residue X_IV_ and residue X_IV+1_. In the second row of the table, the interactions from the carboxylesterase SshEstI (SCOP family #2, PDB ID:3WJ1_A) are listed, corresponding to the interactions that are illustrated in Fig 2. In four ABH fold families [SCOP families #12 (PDB ID:1MJ5_A), #23, #27 and #29], residue X_IV_ is the catalytic acid residue. SCOP family #12 is represented by two structures in order to properly reflect the local structural variations in ABH fold enzymes that have their catalytic acid at the canonical position (i.e. at the turn that follows strand β7, e.g. structure PDB ID:1B6G_A) or at the alternate position (i.e. at the position of residue X_IV_, e.g. structure PDB ID:1MJ5_A). Alternative types of contacts to those described as conserved occur in a few representative structures, for example in ABH fold enzyme families of Epoxide hydrolase (SCOP family #18), Atu1826-like (SCOP family #36) and PHB depolymerase-like (SCOP family #37). Values in parentheses correspond to the distances of the hydrogen bond to the hydrogen atom.

SCOP family # / SCOP family name	PDB ID Reference	CA/Nucleophile–O/X_IV_	O/Nucleophile–CA/X_IV+1_	Sc/(Nucleophile+4)–O/X_IV+2_
2. Carboxylesterase	3WJ1_A [13]	CA/S151-O/Y177 3.2 (2.2)	O/S151-CA/P178 3.6 (2.6)	CB/N155-O/A179 3.0 (2.2) ND2/N155-O/A179 3.0
1. Acetylcholinesterase-like	1QE3_A [82]	CA/S189-O/S215 3.2 (2.2)	O/S189-CA/G216 3.8 (2.8)	CA/M193-O/A217 3.2 (2.2) CG/M193-O/A217 3.4 (2.6)
2. Carboxylesterase	1LZL_A [83]	CA/S160-O/I188 3.0 (2.0)	O/S160-CA/P189 3.6 (2.6)	CA/G164-O/E190 3.1 (2.3)
3. Mycobacterial antigens	1DQZ_A [84]	CA/S124-O/S148 3.3 (2.3)	O/S124-CA/G149 3.5 (2.4)	CA/G128-O/F150 4.6 (3.7)
4. Hypothetical protein TT1662	1UFO_A [85]	CA/S113-O/I136 3.4 (2.4)	O/S113-CA/G137 4.4 (3.5)	CA/F117-O/S138 3.5 (2.7) CD1/F117-O/S138 3.4 (2.4)
5. PepX catalytic domain-like	3PUI_A [86]	CA/S117-O/M141 3.2 (2.2)	O/S117-CA/A142 4.5 (3.7)	CA/V121-O/S143 4.0 (3.1) CG1/V121-O/S143 3.8 (2.9) CG2/V121-O/S143 3.6 (2.6)
6. Prolyl oligopeptidase, C-terminal domain	1H2W_A [87]	CA/S554-O/V578 3.1 (2.1)	O/S554-CA/G579 3.4 (2.4)	CB/L558-O/V580 3.4 (2.5) CD1/L558-O/V580 3.6 (2.7)
7. DPP6 catalytic domain-like	1ORV_A [88]	CA/S630-O/A654 3.3 (2.3)	O/S630-CA/P655 3.6 (2.5)	CB/Y634-O/V656 3.4 (2.7) CD1/Y634-O/V656 3.1 (2.1)
8. Serine carboxypeptidase-like	3SC2_A [89]	CA/S146-O/N176 3.1 (2.2)	O/S146-CA/G177 3.2 (2.6)	CA/G149-O/L178 3.3 (2.4)
9. Gastric lipase	1HLG_A [90]	CA/S153-O/A180 3.4 (2.5)	O/S153-CA/P181 4.9 (4.1)	CB/T157-O/V182 2.9 (1.9)
10. Proline aminopeptidase-like	1MTZ_A [91]	CA/S105-O/G129 3.0 (2.1)	O/S105-CA/G130 3.7 (2.7)	CB/A109-O/L131 3.3 (2.6)
11. Acetyl xylan esterase-like	1L7A_A [92]	CA/S181-O/Y204 3.2 (2.3)	O/S181-CA/P205 3.6 (2.7)	CA/G185-O/Y206 3.4 (2.7)
12. Haloalkane dehalogenase	1B6G_A [93]	N/D124-O/N148 2.8	O/D124-CB/A149 4.1 (3.5)	CA/F128-O/C150 3.6 (2.6) CD1/F128-O/C150 3.3 (2.3)
	1MJ5_A [94]	N/D108-O/E132 3.0	O/D108-CA/A133 4.6 (3.8)	CA/A112-O/I134 4.7 (4.0)
13. Dienelactone hydrolase	1ZI9_A [95]	CA/S123-O/Y145 3.3 (2.2)	O/S123-CA/G146 3.4 (2.3)	CB/A127-O/V147 3.5 (2.7)
14. Carbon-carbon bond hydrolase	2OG1_A [96]	CA/S112-O/G136 3.2 (2.4)	O/S112-CA/P137 3.7 (2.8)	CB/A116-O/G138 3.6 (2.6)
15. Biotin biosynthesis protein BioH	4ETW_A [39]	CA/A82-O/A106 3.4 (2.6)	O/A82-OG/S107 2.6	CD2/L86-O/S108 3.6 (2.8)
16. Aclacinomycin methylesterase RdmC	1Q0R_A [97]	CA/S102-O/L126 3.2 (2.1)	O/S102-CA/G127 4.0 (3.3)	OG1/T106-O/G128 4.4
17. Carboxylesterase/lipase	4DIU_A [98]	CA/S93-O/C115 3.2 (2.2)	O/S93-CA/A116 3.5 (2.6)	CG2/V97-O/P117 3.7 (3.1)
18. Epoxide hydrolase	1QO7_A [99]	CA/D192-CD2/L215 3.5	O/D192-CD2/L215 3.9 (2.9)	CB/S195-O/L215 3.5 (2.8)
19. Haloperoxidase	1BRT_A [100]	CA/S98-O/A123 3.1 (2.2)	O/S98-CA/S124 4.3 (3.2)	OG1/T101-N/L125 3.2
20. Thioesterases	1EI9_A [101]	CA/S115-O/G140 3.6 (2.5)	O/S115-CA/G141 3.4 (2.4)	OE1/Q119-N/Q142 2.8 ND2/Q119-O/Q142 3.8
21. Carboxylesterase/ thioesterase 1	1FJ2_A [102]	CA/S114-O/S138 3.2 (2.3)	O/S114-CB/C139 3.1 (2.6)	CA/A118-O/W140 3.1 (2.3)
22. Ccg1/TafII250-interacting factor B (Cib)	1IMJ_A [103]	CA/S111-O/A135 3.3 (2.3)	O/S111-CA/P136 3.4 (2.5)	CA/G114-O/I137 3.6 (2.7)
23. A novel bacterial esterase	1QLW_A [104]	CA/S206-O/E230 3.2 (2.3)	O/S206-CA/P231 3.7 (2.8)	O/S206-N/G232 2.9
24. Lipase	1JFR_A [105]	CA/S131-O/T154 3.2 (2.2)	O/S131-CA/G155 3.5 (2.5)	CA/G135-O/W156 3.3 (2.3)
25. Fungal lipases	1TCA_A [106]	CA/S105-O/A132 3.3 (2.4)	O/S105-CA/P133 3.7 (2.6)	CD2/L109-O/D134 4.0 (3.0) CB/L109-OD1/D134 3.8 (3.0)
26. Bacterial lipase	1ISP_A [107]	CA/S77-O/G103 3.3 (2.3)	O/S77-CA/G104 3.4 (2.4)	CB/A81-O/A105 3.3 (2.2)
27. Pancreatic lipase, N-terminal domain	1BU8_A [108]	CA/S152-O/D176 3.4 (2.6)	O/S152-CA/P177 3.7 (2.7)	NE2/H156-O/A178 2.7
28. Hydroxynitrile lyase-like	3C6X_A [109]	CA/S80-O/N104 3.1 (2.4)	O/S80-CA/S105 3.2 (2.5)	CA/G83-O/V106 3.4 (2.5)
29. Thioesterase domain of polypeptide, polyketide and fatty acid synthases	1JMK_C [110]	CA/S80-O/D107 5.5 (4.7)	O/S80-OG/S108 3.1	CB/S84-O/Y109 5.1 (4.1)
30. Cutinase-like	1BS9_A [111]	CA/S90-O/G132 3.4 (2.3)	O/S90-CA/D133 4.0 (2.9)	OE1/E94-CG/P134 3.5 (2.8)
31. YdeN-like	1UXO_A [112]	CA/S71-O/S97 3.6 (2.6)	O/S71-CA/G98 4.7 (3.7)	CB/P75-O/F99 3.6 (2.8) CD/P75-O/F99 3.5 (2.6)
32. Putative serine hydrolase Ydr428c	1VKH_A [113]	CA/S110-O/D151 3.3 (2.4)	O/S110-CA/G152 3.7 (2.7)	OG1/T114-O/I153 2.7 CG2/T114-O/I153 3.3 (2.5)
33. Acylaminoacid-releasing enzyme, C-terminal domain	1VE6_A [114]	CA/S445-O/A469 3.1 (2.2)	O/S445-CA/S470 3.9 (2.8)	CA/Y449-O/V471 3.7 (2.8) CD1/Y449-O/V471 3.3 (2.2)
34. Hypothetical esterase YJL068C	1PV1_A [115]	CA/S161-O/A187 3.1 (2.1)	O/S161-CA/P188 3.6 (2.6)	CB/Y165-O/I189 3.0 (1.9) CD1/Y165-O/I189 3.5 (2.9)
35. Hypothetical protein VC1974	1R3D_A [116]	CA/S91-O/G118 3.4 (2.4)	O/S91-CA/G119 3.7 (2.9)	CG/R95-O/H120 3.4 (2.6) CD/R95-O/H120 3.3 (2.8)
36. Atu1826-like	2I3D_A [117]	CA/S108-O/A131 3.3 (2.2)	O/S108-CA/P132 4.5 (3.4)	W112-Y137 stacking
37. PHB depolymerase-like	2D80_A [118]	CA/S39-O/A64 3.3 (2.5)	O/S39-CA/G65 3.9 (3.2)	Y43-G66 stacking
38. IroE-like	2GZR_A [14]	CA/S189-O/S212 3.2 (2.2)	O/S189-CA/P213 3.4 (2.4)	CD1/L193-O/S214 3.7 (2.6) CD2/L193-O/S214 3.9 (3.0)
40. O-acetyltransferase	2B61_A [119]	CA/S143-O/C167 3.5 (2.6)	O/S143-CB/S168 3.2 (2.4)	CG/M147-O/S169 3.8 (3.1) CE/M147-O/S169 3.5 (3.1)
41. 2,6-dihydropseudo-oxynicotine hydrolase-like	2JBW_A [120]	CA/S217-O/G240 3.2 (2.2)	O/S217-CA/G241 3.2 (2.2)	CB/N221-O/F242 3.2 (2.5) ND2/N221-O/F242 2.9

**Fig 2:**
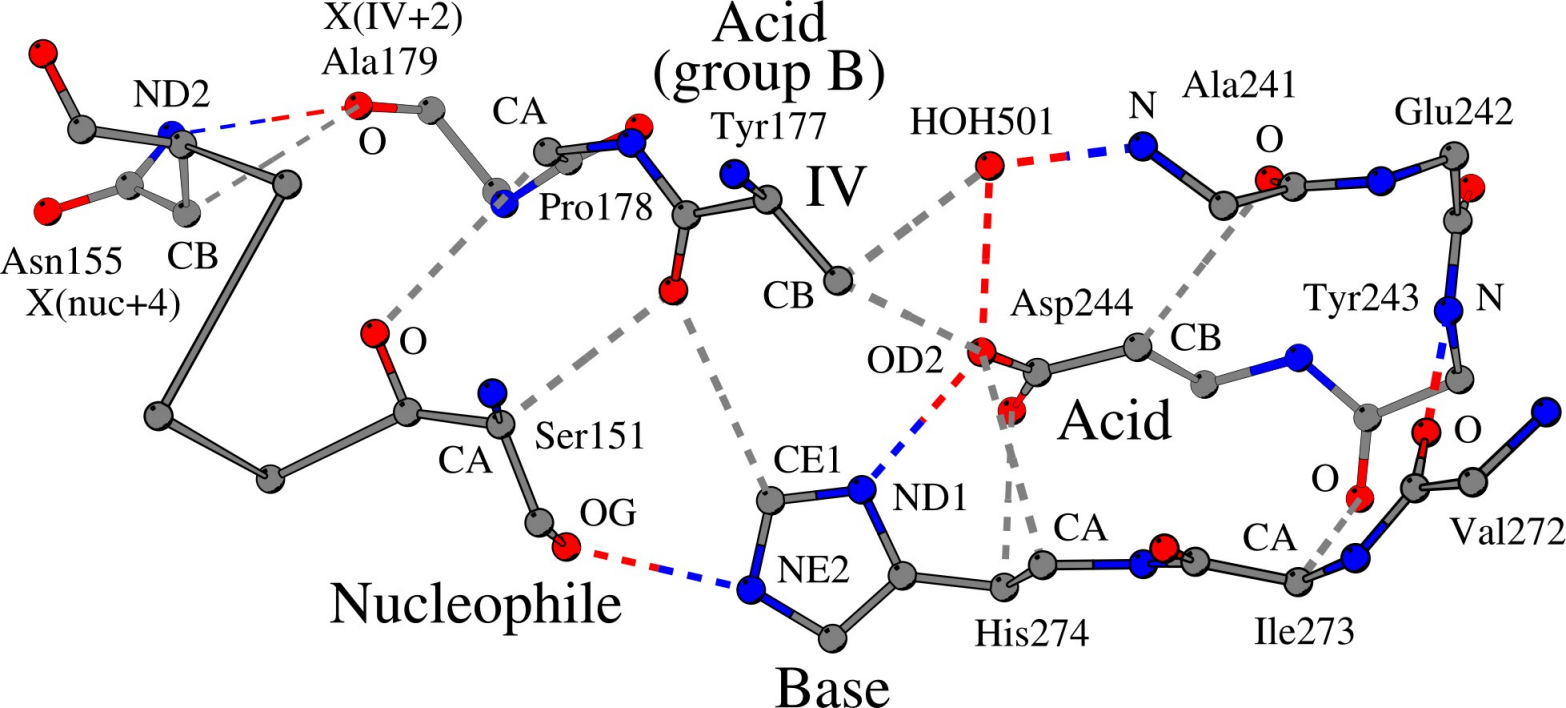
Coordination of the catalytic nucleophile-histidine pair and the catalytic histidine-acid pair in the carboxylesterase SshEstI (PDB ID:3WJ1). The catalytic nucleophile (“Nucleophile”, Ser151 in SshEstI) is hydrogen bonded (OG/Ser151 –NE2/His274) to the catalytic histidine (“Base”, His274 in SshEstI) as part of the standard interaction network of the residues of the catalytic machinery. However, three additional conserved interactions ensure the fine turning of the two catalytic residues next to each other: The main-chain oxygen atom of Tyr177 that is located at the C-terminus of strand β6 (termed and shown as “IV”) forms two weak hydrogen bonds: one with the catalytic nucleophile (O/Tyr177 –CA/Ser151) and another with the catalytic histidine (O/Tyr177 –CE1/His274); a third contact (O/Ser151 –CA/Pro178) is formed between the catalytic nucleophile and Pro178 (located ahead of position IV) and coordinates the catalytic nucleophile. Thus, these three interactions support the optimal arrangement of the catalytic nucleophile-histidine pair. The catalytic histidine (His274) interacts (ND1/His274 –OD2/Asp244) with the catalytic acid (“Acid”, Asp244 in SshEstI) and is further supported by two weak hydrogen bonds: OD2/Asp244 –CA/His274 and OD1/Asp244 –CB/His274. Interactions of the catalytic acid zone that are associated with the coordination of the catalytic histidine and other contacts located nearby the catalytic site are shown. Gray dashed lines, weak hydrogen bonds; colored dashed lines, standard hydrogen bonds.

In the carboxylesterase SshEstI [13] (Fig 2), for example, the catalytic nucleophile Ser151 forms two weak hydrogen bonds with its neighboring residues: one bond with Tyr177_IV_ (CA/Ser151 –O/Tyr177) and a second bond with Pro178_IV+1_ (O/Ser151 –CA/Pro178). The former contact, the weak hydrogen bond between the catalytic nucleophile (Ser151) and the residue at position IV (Tyr177), coordinates the catalytic nucleophile next to the catalytic histidine, because the main-chain oxygen atom of Tyr177_IV_ also forms the already-reported [10], conserved interaction with the catalytic histidine (O/Tyr177 –CE1/His274). The second weak hydrogen bond observed between the catalytic nucleophile and residue X_IV+1_ is involved in the coordination of the catalytic nucleophile.

The two weak hydrogen bonds between the catalytic nucleophile with residue X_IV_ and residue X_IV+1_ are absolutely conserved in all 40 ABH fold enzyme families (Table 1). Because both interactions involve main-chain atoms, the identified hydrogen-bonding scheme is not affected when the catalytic acid occurs at the C-terminus of strand β6, while it also remains intact regardless of the type of amino acids that occur at positions IV and IV+1. Specifically, residue X_IV_ is often occupied by a small amino acid (glycine in 7 of 40 ABH fold enzyme families) or a hydrophobic amino acid (16 ABH fold families), however bulky side chains such as tyrosine (2 ABH fold families) or arginine (2 ABH fold families) are tolerated at position IV, too. Residue X_IV+1_ is much more conserved than residue X_IV_, and is most commonly a glycine (16 of 40 ABH fold families) or a proline (12 ABH fold families).

Besides the two weak hydrogen bonds that coordinate the catalytic nucleophile-histidine pair, in our search for structural elements at the C-termini of strand β5 and strand β6, we have identified an additional conserved weak hydrogen bond that occurs in the vast majority of ABH enzyme families (Table 1). While not involved in the coordination of the catalytic residues, the contact CB/N155 –O/A179 in SshEstI (Fig 2) links two residues that are situated at the core of the active site: the residue that is located 4 positions after the catalytic nucleophile (Asn155_nuc+4_ in SshEstI) with the residue that is located at position IV+2 (Ala179_IV+2_ in SshEstI). Both residues are hydrophobic or aromatic in approximately 30 ABH enzyme families (Table 1).

The two weak hydrogen bonds formed between the catalytic nucleophile and residue X_IV_ and residue X_IV+1_ clearly serve to coordinate the catalytic nucleophile next to the catalytic histidine. The optimal arrangement of the catalytic nucleophile-histidine pair is additionally supported by the interaction of the nucleophile zone and the oxyanion zone with the loop fragment following the catalytic histidine, which helps stabilizing the flexible histidine loop located “above” the β-sheet. Likewise, on the other side of the catalytic machinery, the catalytic acid zone interacts with the loop fragment that precedes the catalytic histidine, fixing it close to the catalytic acid. Subsequently, we have performed a similar search for structural elements that coordinate the catalytic histidine next to the catalytic acid in the vicinity of the catalytic acid zone and, below, we show that conserved elements that contribute to the optimal arrangement of the catalytic acid-histidine pair.

### Coordination of the catalytic acid next to the catalytic histidine

The interaction of the catalytic acid with the catalytic histidine takes place around the catalytic acid zone, near the C-termini of strand β6 and strand β7 [8], with the turn that follows strand β7 accommodating the catalytic acid residue in the majority of ABH fold enzyme families. As mentioned earlier, the catalytic acid zone helps stabilize the catalytic histidine loop, because the residue that precedes the catalytic acid interacts with the residue that is located two positions prior to the catalytic histidine.

Here, we have revisited the catalytic acid zone in order to search for structural elements that coordinate the catalytic acid next to the catalytic histidine. The comparative analysis of the active sites of enzymes from 40 ABH fold families has revealed four conserved interactions that fix the catalytic acid and the catalytic histidine in an optimal arrangement. Specifically, we have found two weak hydrogen bonds that are formed between the catalytic acid and the catalytic histidine (S1A Table), and two additional interactions that involve the imidazole ring of the catalytic histidine (column I set, S1B Table).

**Fig 3:**
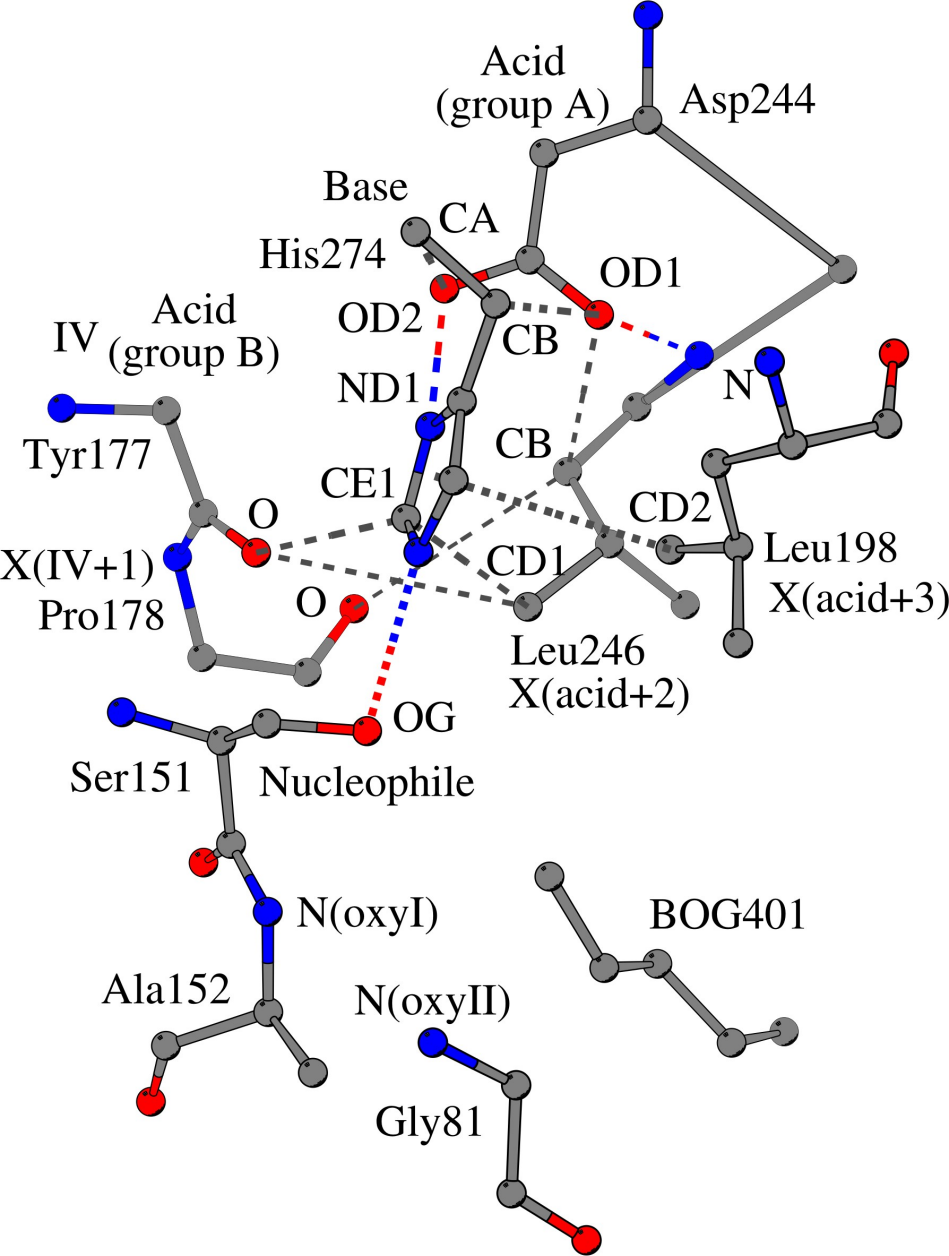
Coordination of the imidazole ring of the catalytic histidine in the carboxylesterase SshEstI (PDB ID:3WJ1). The optimal arrangement of catalytic residues requires the proper positioning of the imidazole ring of the catalytic histidine (“Base”, His274 in SshEstI) relative to the side chains of the catalytic nucleophile (“Nucleophile”, Ser151 in SshEstI) and the catalytic acid (“Acid”, Asp244 in SshEstI). The catalytic histidine is anchored to the β-sheet through its contact with Tyr177 (position IV) and is directly coordinated by the catalytic acid Asp244. The imidazole ring of the catalytic histidine is coordinated by two interactions: a hydrophobic interaction (CD1/Leu246 –CE1/His274) with Leu246 (X(acid+2)), which is located two sequence positions after the catalytic acid, and a CH–π interaction (CD2/Leu198 –π/His274) with Leu198 (X(acid+3)). The residues at the C-terminal end of strand β6, Tyr177 (IV) and Pro178 (IV+1), also interact with Leu246, which is situated at the turn that accommodates the catalytic acid. The residues, “OxyI” and “OxyII”, which help form the oxyanion hole, and the bound ligand “BOG401” of carboxylesterase SshEstI are indicated. Gray dashed lines represent weak hydrogen bonds; colored dashed lines, standard hydrogen bonds.

In SshEstI [13] for example (Fig 2), the catalytic acid Asp244 is coordinated next to the catalytic histidine by two weak hydrogen bonds: the first interaction is formed between the side-chain oxygen atom of Asp244 and the CA atom of His274 (CA/His274 –OD2/Asp244), while the second interaction links the other side-chain oxygen atom of Asp244 with the CB atom of His274 (CB/His274 –OD1/Asp244). The imidazole ring of His274 forms two contacts with two other neighboring residues (Fig 3). The first contact is a hydrophobic interaction between the catalytic His274 and Leu246 (CD1/Leu246 –CE1/His274); Leu246 is located at the catalytic acid turn, two sequence positions after the catalytic acid. The second contact is a CH–π interaction between His274 and Leu198 (CD2/Leu198 –π/His274); residue Leu198 is located at a flexible loop opposite to the β-sheet and is part of the helix-turn-strand supersecondary structure that connects strand β6 with strand β7.

The two weak hydrogen bonds formed between the catalytic acid and the catalytic histidine are observed in almost all ABH fold enzyme families regardless of the position of the catalytic acid (S1A Table), with the single exception of the hydrolase IroE from *Escherichia coli* (PDB ID:2GZR), which is suggested to have an atypical catalytic nucleophile-base dyad [14]. The interactions that involve the imidazole ring of the catalytic histidine are also recurrent in the 40 ABH fold enzyme families (column I set, S1B Table). The hydrophobic interaction is conserved in 32 ABH fold families; in 22 ABH fold families the interaction involves the residue that is located three positions after the catalytic acid (here termed residue X_acid+3_), while in 7 ABH fold families the residue is located two positions after the catalytic acid (which we hereafter refer to as residue X_acid+2_), for example Leu246_acid+2_ in SshEstI. The CH–π interaction is conserved in 37 ABH fold families, with residue X_acid+2_ involved in the CH–π interaction with the imidazole ring in 29 ABH fold families, while in the remaining cases, the catalytic histidine interacts with some other neighboring residue, such as Leu198 in SshEstI. In the 22 of 40 ABH fold enzyme families, the imidazole ring of the catalytic histidine is simultaneously coordinated by a hydrophobic interaction with residue X_acid+3_ and a CH–π interaction with residue X_acid+2_, while in 6 ABH fold enzyme families the imidazole ring is coordinated in a similar way to that observed in the reference structure SshEstI, which we have described in the previous paragraph.

Besides their role in the coordination of the catalytic residues, residue X_acid+2_, residue X_IV_ and residue X_IV+1_ are linked through a separate hydrogen-bonding network (Fig 3), with residue X_acid+2_ (Leu246 in SshEstI) forming one weak hydrogen bond with residue X_IV_ (Tyr177 in SshEstI) and another weak hydrogen bond with residue X_IV+1_ (Pro178 in SshEstI). This interaction pattern is conserved in 35 of 40 ABH enzyme families (column II set, S1B Table).

In total, six conserved interactions coordinate the catalytic histidine next to the catalytic nucleophile and the catalytic acid at the active site of ABH fold enzymes. The conserved, structural elements that comprise the catalytic acid zone, the nucleophile zone and the oxyanion zone, together with the newly identified structural elements that coordinate the catalytic histidine, make up the catalytic structural core of the ABH fold enzymes. After perceiving the catalytic core as a composition of conserved structural elements, we have furthered our analysis to explore the role of the conserved structural core in ligand-bound ABH structures. As we show below, it appears that several elements of the conserved structural core not only contribute to the optimal arrangement of residues of the catalytic mechanism, but also engage in ligand binding.

### Elements of the conserved, catalytic structural core of ABH fold enzymes participate in the binding of the ligand

**Fig 4:**
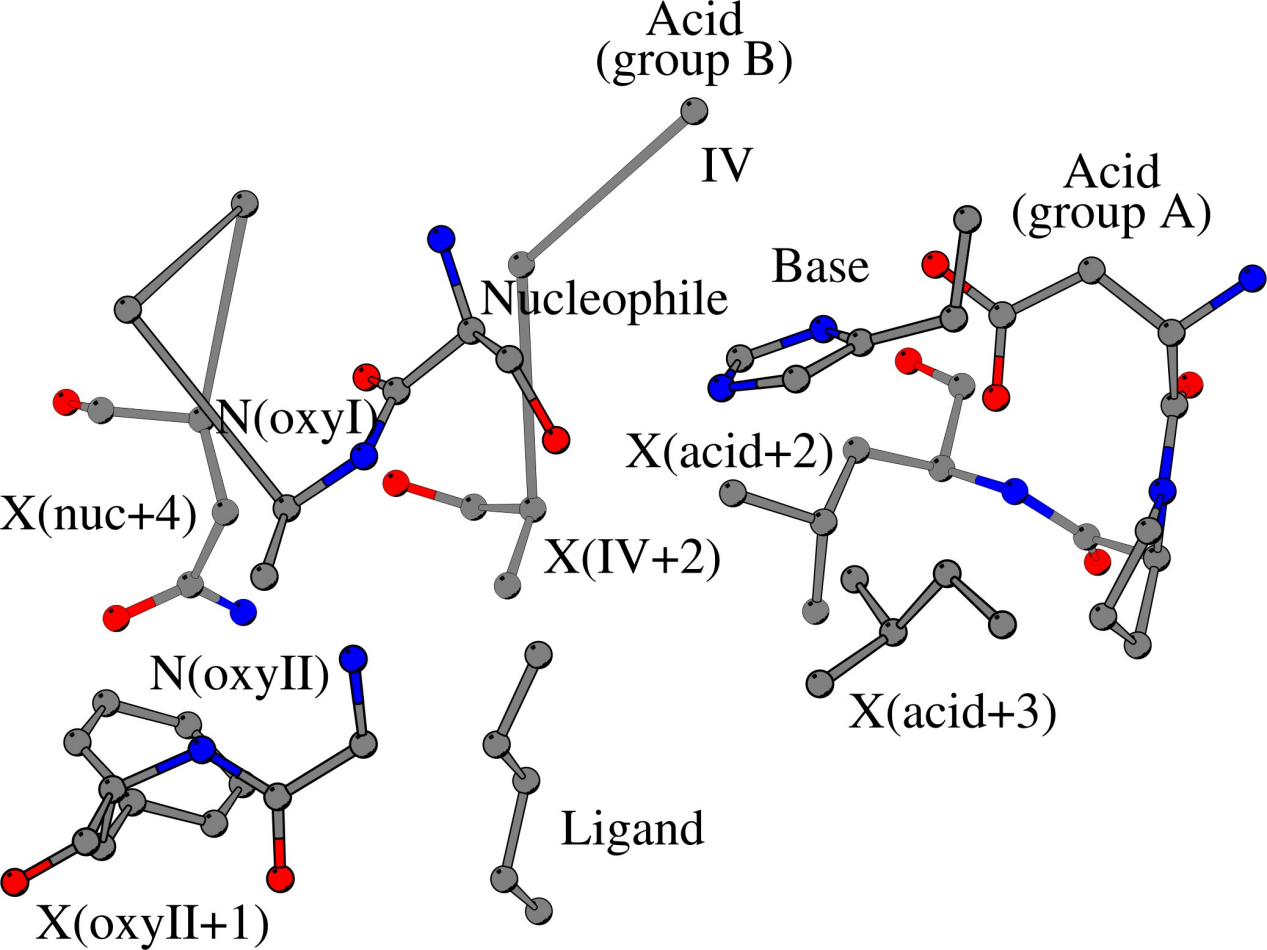
Structural elements at the active site of the ABH fold enzymes surround the bound ligand. Recognized substrate molecules (shown as “Ligand”) are bound to the active sites of ABH fold enzymes in order to be hydrolyzed. Key residues of the catalytic machinery, such as the catalytic histidine (“Base”), the catalytic nucleophile (“Nucleophile”) and the residues that help form the oxyanion hole (“oxyI” and “oxyII”) often interact with the bound substrate; the catalytic acid, either at its canonical position (at the turn that follows strand β7, “Acid (group A)”) or at its alternate position (at the C-terminal end of strand β6, “Acid (group B)”) does not participate in ligand binding. Several residues, which belong to the structural core of ABH fold enzymes, participate both in the coordination of the residues of the catalytic machinery and in ligand binding, including the residues that are located two and three sequence positions after the catalytic acid, illustrated as “X(acid+2)” and “X(acid+3)” respectively, and the residue at the C-terminus of strand β6. Other residues, usually comprising the substrate binding core, are illustrated as “X(IV+2)” for the residue that is located two positions after the C-terminus of strand β6, “X(oxyII+1)” for the residue that follows the second residue that helps form the oxyanion hole, and “X(nuc+4)” for the residue that is located four positions after the catalytic nucleophile.

Here, we define the catalytic structural core to be the geometry that is located at the active site of ABH fold enzymes and consists of the conserved structural elements that directly and indirectly coordinate the key units of the catalytic mechanism. In detail, the catalytic acid zone, the nucleophile zone, the oxyanion zone and the structural elements that are involved in the coordination of the catalytic histidine form a distinct interaction network that is common among the ABH fold enzyme families. The catalytic structural core extends through the whole area of the active site, which consequently raises the question of how the conserved geometry of the catalytic core correlates with the binding sites of ABH fold enzymes. Because ligand binding is highly dependent on both the structure of the ligand and the amino-acid variations of the protein interior, the comparison of the binding sites of the functionally diverse ABH fold enzymes appears to be counterintuitive. However, our attempt to compare the binding sites of ligand-bound ABH fold enzymes from 28 ABH fold enzyme families based on the conserved structural elements of the catalytic core has shown that the conserved structural elements of catalytic core often coincide with the residues that are involved in the binding of ligands, regardless of the structure of the ligand or the type of amino-acids that line the active sites of the ABH fold enzymes (Fig 4).

Starting with the catalytic acid zone, we have found that the catalytic acid does not participate in ligand binding (column Catalytic acid zone, Table 2). However, residue X_acid+2_ (Leu246 in SshEstI) and residue X_acid+3_, which coordinate the imidazole ring of the catalytic histidine, routinely form contacts with the ligand in the majority of the ligand-bound ABH structures (column Catalytic acid zone, Table 2); the same applies for residues that are involved in the coordination of the imidazole ring but do not occur at conserved positions, such as Leu198 in SshEstI. Similarly, the catalytic histidine frequently interacts with the ligand (under Catalytic base loop in Table 2), sometimes accompanied by the residue that follows in sequence.

**Table 2:** Elements of the conserved catalytic structural core are involved in ligand binding in 28 ABH fold enzyme families. In ligand-bound represenative structures, the recognized substrate molecule (Ligand) frequently interacts with residues that belong to the conserved structural organizations of the ABH fold, including the oxyanion zone (Oxyanion zone), the nucleophile zone (Nucleophile zone), the C-terminal end of strand β6 (Strand β6) and the catalytic acid zone (Catalytic acid zone); residues from the loop that accommodates the catalytic histidine (Catalytic base loop) can also participate in ligand binding. Two ABH fold enzyme families (SCOP family #15 and SCOP family #18) are represented by two structures, each bound to a different substrate, while one ABH fold enzyme family (SCOP family #12) is represented by three representative structures that are bound to three different substrates. The first entry is for carboxylesterase SshEstI (SCOP family #2, PDB ID:3WJ1_A). Residues that are in bold signify that the specific residue does not belong to the conserved catalytic core, but are adjacent to it. Fields of the table that contain the entry “None” indicate that the corresponding geometry does not participate in ligand binding.

SCOP family # / SCOP family name	PDB ID Reference	Ligand	Oxyanion zone	Nucleophile zone	Strand β6	Catalytic acid zone	Catalytic base loop
2. Carboxylesterase	3WJ1_A [13]	BOG 401_A	G81, F82	S151, A152	**A179**	L198, L246	None
2. Carboxylesterase	1LZK_A [83]	CAC 500_A	G87, G88, G89	Q159, S160, A161	**E190**	W209, L262	H290
3. Mycobacterial antigens	1DQY_A [84]	DEP 401_A	G39, L40, R41	L123, S124, M125	**F150**	L227	None
5. PepX catalytic domain-like	3I2K_A [121]	DBC 591_A	Y44	S117, Y118, **V121**	None	F261, L407	H287
6. Prolyl oligopeptidase, C-terminal domain	2XDW_A [122]	PHQ 791_P PRO 792_P YCP 793_P	Y473, F476, I478	S554, N555	**V580**	R643, V644	H680
7. DPP6 catalytic domain-like	2AJ8_A [123]	SC3 1601_A	V546, Y547	W629, S630, Y631	**V656**	N710, V711	H740
8. Serine carboxypeptidase-like	1WHT_A, B [124]	BZS 430_A	N51, G52, G53, C56	E145, S146, Y147	None	Y239, V340	H397, E398
10. Proline aminopeptidase-like	1MT3_A [91]	MES 200_A	G36, G37	S105, Y106	**L131**	E245, V246	H271, L272
11. Acetyl xylan esterase-like	1ODT_C [125]	ACT 1318_C	G90, Y91, A93	A181, Q182	**Y206**	V271	H298
12. Haloalkane dehalogenase	1BE0_A [126]	ACY 401_A	E56	D124, W125 **F128**	None	L262, L263	H289
	2DHD_A [127]	0AK 124_A	G55, E56	Q123, W125, G127, **F128**	N148, A149	L262, L263	H289
	2BFN_A [128]	D2P 1297_A	N38	D108	**I134**	L177, L248	H272, F273
13. Dienelactone hydrolase	1ZJ4_A [95]	SEB 123_A	D36, I37, F38	Y122, L124, G126, **A127**	Y144, Y145, **V147**	F173, V174	H202, S203
14. Carbon-carbon bond hydrolase	2RHW_A [129]	C0E 288_A	G41, G42, G43, A46	N111, A112, M113	**G138**, G139	F239, V240	H265, W266
15. Biotin biosynthesis protein BioH	1M33_A [130]	3OH 300_A	G21, W22, L24	W81, S82, L83	None	L209	H235
	4ETW_A [39]	ZMK 600_B	G21, W22, L24	W81, S82, L83	None	L209	H235
16. Aclacinomycin methylesterase RdmC	1Q0R_A [97]	AKT 600_A	G32, N33	S102, M103, **T106**	L126, G127, **G128**, G129	I250, A251	H276
17. Carboxylesterase/lipase	1TQH_A [131]	4PA 701_A	G24, F25, T26, G27	L93, S94, L95	**P118**	M195, I196	H223, V224
18. Epoxide hydrolase	3G02_A [132]	FMT 408_A	W117	D192	None	W284, L349	No
	3G0I_A [132]	VPR 1_A	W117	D192, I193, **F196**	**L215**	W284, L349	H374
19. Haloperoxidase	1A8U_A [100]	BEZ 295_A	G31, F32	F97, S98, M99	**L125**	T230, L231	H257
20. Thioesterases	1EXW_A [133]	HDS 430_A	G40, M41	S115, Q116	**Q142**	I235, V236	H289, L290
21. Carboxylesterase/ thioesterase 1	5SYM_A [134]	71Q 301_A	L30	S119, Q120	**W145**, L146	L176, V177	None
22. Ccg1/TafII250-interacting factor B (Cib)	1IMJ_A [103]	SO4 211_A	G40, I41, R42, F43	S111, L112	None	M164	H188, P189, Y191
23. A novel bacterial esterase	2WKW_A [104]	W22 577_A	G70, C71, C72, L73	H205, S206, Q207	**G232**	F145, W262	H298
25. Fungal lipases	5A71_A [135]	IPA 1319_A	T40	S105	**D134**	I189, V190	H224
26. Bacterial lipase	1R50_A [136]	SIL 277_A	G11, I12, G13, G14	H76, S77, M78	**A105**	I135, V136	H156, I157, L160
27. Pancreatic lipase, N-terminal domain	1LPB_B [137]	MUP 901_B	G76, F77, D79	H151, S152, L153	**A178**	F215	H263, L264
28. Hydroxynitrile lyase-like	3C70_A [109]	SCN 1001_A	T11, I12, H14	S80, C81	None	I209, F210	H235, K236
30. Cutinase-like	1G66_A [138]	SO4 210_A	E12, T13	S90, Q91	**P134**	Y177	H187, Q188
33. Acylaminoacid-releasing enzyme, C-terminal domain	4RE5_A [139]	Y3A 601_A	G368, G369, P370, F371	S445, Y446	**V471**	R526, T527	H556
34. Hypothetical esterase YJL068C	3C6B_A [115]	SDP 161_A	G57, L58, C60	H160, M162, G164, **Y165**	F186, A187, P188, **I189**	F243, L248	H276, Y278
37. PHB depolymerase-like	2D81_A [118]	RB3 451_A	C250, L251	A39, S40, **Y43**	None	T123, V124	H155

The search for ligand-binding interactions near the C-terminus of strand β6 has shown that residue X_IV_ (Tyr177 in SshEstI) and residue X_IV+1_ (Pro178 in SshEstI) occasionally participate in ligand binding, while residue X_IV+2_ (Ala179 in SshEstI) regularly interacts with the ligand (column Strand β6, Table 2).

In the vicinity of the nucleophile zone and the oxyanion zone, we have found several residues that are involved in ligand binding, with some of them belonging to the conserved catalytic core and others being located at a fixed distance from conserved structural elements of the catalytic core (column Oxyanion zone and column Nucleophile zone, Table 2). Within the nucleophile zone, residues of the nucleophile elbow that interact with the ligand in the majority of ligand-bound ABH structures (column Nucleophile zone, Table 2) include the catalytic nucleophile and the following residue (one of the two oxyanion hole-forming residues). The other residues of the nucleophile elbow rarely participate in ligand binding, except for the residue prior to the catalytic nucleophile. Because of its location at the active site, the residue that is situated four sequence positions after the catalytic nucleophile (Asn155_nuc+4_ in SshEstI) can interact with the ligand, too. Within the oxyanion zone, the second residue of the oxyanion hole commonly binds the ligand (column Oxyanion zone, Table 2), with the preceding and following residues having a major involvement in ligand binding in general.

Most of the 28 sampled ABH fold enzyme families are represented by a single ligand-bound structure, however for three of the 28 ABH fold families, we have found more than one ligand-bound structure. Thus, we have obtained three ligand-bound structures for the ABH fold enzyme family of haloalkane dehalogenases (SCOP family #12), two ligand-bound structures for the ABH fold enzyme family of biotin biosynthesis protein BioH (SCOP family #15), and two ligand-bound structures for the ABH fold enzyme family of epoxide hydrolases (SCOP family #18). The comparison of the ligand-binding sites in each of the three ABH fold families has shown that, regardless of the structure of ligand, the residues that are involved in the coordination of the imidazole ring of the catalytic histidine (Leu246 and Leu198 in SshEstI) consistently interact with the ligand too; the same applies for the second residue of the oxyanion hole that repeatedly forms contacts with the ligand.

To sum up, by comparing the active sites of ligand-bound ABH fold enzymes, we have found that the role of the catalytic structural core is not limited to the coordination of the residues of the catalytic mechanism, but rather, many residues of the conserved structural core also belong to the ligand-binding site. Thus, residues that already have a critical role in the structural integrity of the catalytic mechanism, appear to be involved in the binding of the ligand, with the residues that coordinate the imidazole ring of the catalytic histidine and the second residue of the oxyanion hole being the most common sites of interaction between the conserved structural core and the bound ligand. In the Discussion section, below, we analyze the role of the novel structural elements that are involved in the optimal arrangement of the catalytic triad, and we review their contribution to the conserved geometry of the structural core and the enzymatic activity of ABH fold enzymes.

## Discussion

The focal point of this study is the identification of conserved elements that determine the structural arrangement of the catalytic triad in the ABH fold, aiming to complete the description of the catalytic structural core which was initiated in our previous studies [8, 9]. Specifically, we have earlier introduced three conserved and planar structural organizations that belong to the active site of ABH fold enzymes: the “catalytic acid zone”, the “nucleophile zone” and the “oxyanion zone”, which coordinate the catalytic acid, nucleophile and the residues of the oxyanion hole respectively, and help to fix the catalytic histidine loop over the β-sheet, placing the catalytic histidine close to the other residues of the catalytic triad. Nonetheless, the identification of the three conserved structural organizations only partially explains how the catalytic residues are properly arranged next to each other, because until now there has been insufficient information about the coordination of the catalytic histidine.

Therefore, in this comprehensive analysis, we have explored the active sites of ABH fold enzymes, looking for structural elements that coordinate the residues of the catalytic triad with respect to each other. Our research has been narrowed down to the areas around the catalytic nucleophile-histidine pair and the catalytic acid-histidine pair, where we have identified several structural elements which participate in the optimal arrangement of the residues of the catalytic mechanism. By comparing ligand-bound ABH structures, we have found that conserved structural elements of the catalytic core also engage in ligand binding. Thus, in this section, we discuss our findings and suggest that the conserved, structural elements at the active site of ABH fold enzymes have versatile roles that extend from the coordination of the catalytic residues and the structural integrity of the catalytic mechanism to the binding of ligands and affecting other properties of the enzymatic activity.

### Six conserved interactions coordinate the catalytic histidine next to the catalytic acid and the catalytic nucleophile, and are proven to be critical components for the overall structural stability of the catalytic mechanism

The main concern of this study is the coordination of the catalytic histidine at the active site of ABH fold enzymes. Because the catalytic histidine is located at a flexible loop, it cannot be coordinated by a similar closed structural organization, as in the cases of the catalytic acid, the catalytic nucleophile and the oxyanion hole. Moreover, in a few ABH fold enzymes it has been observed that when the enzyme is inactive, the catalytic histidine is displaced from the catalytic site, but upon ligand binding, the catalytic mechanism is readily re-assembled and the enzyme is again capable of functioning [15–17], which suggests that the flexibility of the histidine loop is also utilized for the regulation of the enzymatic activity. Instead of a closed structural organization, the comparative analysis of active sites from 40 ABH fold enzyme families has shown that the coordination of the catalytic histidine next to the catalytic nucleophile and the catalytic acid is achieved by six conserved interactions from five neighboring residues that coordinate the catalytic histidine, as reviewed below.

Located at the C-terminus of strand β6, at a sharp turn that is suggested to be critical for the position of the residues of the catalytic triad [18–20], residue X_IV_ and residue X_IV+1_ coordinate the catalytic nucleophile-histidine pair. Specifically, residue X_IV_ uses its main-chain oxygen atom to interact with both the catalytic histidine and the catalytic nucleophile, fixing the side chain of the catalytic histidine close to the side chain of the catalytic nucleophile. Residue X_IV+1_ interacts with the main-chain oxygen atom of the catalytic nucleophile, and supports the optimal arrangement of the catalytic nucleophile-histidine pair, through the coordination of the catalytic nucleophile.

In the catalytic acid-histidine pair, it appears that the catalytic acid plays a critical role in the coordination of the catalytic histidine because its side-chain oxygen atoms form two weak hydrogen bonds with the CA and CB atoms of the catalytic histidine. The imidazole ring of the catalytic histidine is further coordinated by nearby residues, usually through two contacts. The first contact is a hydrophobic interaction that takes place between the imidazole ring and residue X_acid+3_ (or in a few cases, with residue X_acid+2_ instead). The second interaction, a CH–π interaction, usually involves the side chain of residue X_acid+2_ or some other residue that stands opposite to the β-sheet and at interacting distance with the imidazole ring.

The findings of this research provide additional information about the role of the catalytic acid zone. The two weak hydrogen bonds between the catalytic histidine and the catalytic acid make the catalytic acid zone directly responsible for the coordination of the catalytic histidine, in addition to the contribution of the catalytic acid zone to the stabilization of the histidine loop. Moreover, in the general vicinity of the catalytic acid zone, residue X_acid+2_ is noteworthy for its two-fold contribution in the coordination of the catalytic histidine, because it not only coordinates the imidazole ring of the catalytic histidine, but X_acid+2_ is also part of the Asx-motif, which is suggested to aid the correct orientation of the catalytic histidine in the ABH enzymes that have a catalytic aspartic acid after strand β7 [21]. In particular, the Asx-motif occurs at the turn after strand β7 and includes the catalytic aspartic acid, residue X_acid+2_, and frequently residue X_acid+3_, which together form a hydrogen-bonding network that connects the side-chain oxygen of the catalytic acid with the main-chain nitrogen atoms of residue X_acid+2_ and residue X_acid+3_; the same side-chain oxygen of the catalytic acid interacts with the catalytic histidine, and thus, the Asx-motif is thought to help stabilize the catalytic histidine. Remarkably, we have found that residue X_acid+2_ regularly interacts with both residue X_IV_ and residue X_IV+1_, which indicates that the conserved structural elements that surround the residues of the catalytic triad are joined together in a circular manner.

All things considered, our observations suggest that the catalytic triad is coordinated by an extended network of interwoven contacts among neighboring residues that are located at conserved positions on the ABH fold. Residue X_IV_, residue X_IV+1_ and residue X_acid+2_ are prime structural elements of the catalytic core that orchestrate the optimal arrangement of the catalytic triad, with the interactions that involve residue X_IV_, residue X_IV+1_ and residue X_acid+2_ clearly ensuring the structural integrity of the catalytic mechanism in the active site of ABH enzymes.

### The conserved structural elements that line the catalytic structural core of ABH fold enzymes are important for the enzymatic function because they are actively involved in ligand binding and affect other properties of the enzymatic activity

With the identification of the conserved structural elements that coordinate the catalytic histidine next to the catalytic acid and the catalytic nucleophile, and combined with the determination of the conserved geometries that surround the catalytic acid, the catalytic nucleophile and the residues that form the oxyanion hole, we suggest that the sum of the conserved structural elements that we have in all three studies make up the conserved catalytic structural core, which is the minimum common structure of the active site of ABH fold enzymes.

The catalytic structural core is located at the interior of the ABH fold proteins and extends from the area around the catalytic acid to the area around the oxyanion hole. The elements that comprise the catalytic structural core are located at conserved positions across the ABH fold, including the C-termini of strand β3-β7, the N-termini of helix αA and αC, the turn after strand β7 and a part of the flexible loop after strand β8. Considering the striking conservation rate of the catalytic structural core in the 40 ABH fold enzyme families, it seems that the “skeleton” of the active site is largely maintained among the ABH fold enzymes, regardless of the catalytic versatility that characterizes the structural family of alpha/beta-Hydrolases.

Based on this observation, we have explored ligand-bound ABH structures to find out how the conserved structural core correlates with the binding site of ABH fold enzymes, where we have verified that several conserved structural residues of the active site interact with the ligand, and thus, the distinct architecture of the conserved catalytic core also serves ligand binding (Table 2). However, we have observed that ligand binding is also dependent on hydrophobic/aromatic residues that are located before and after the conserved structural residues, such as the residues that precede and follow residue X_acid+2_, residue X_IV+2_ that follows the dipeptide residue X_IV_—residue X_IV+1_, and the residues at the N-terminus of loop_β3→αA_ that follow the oxyanion zone. Therefore, we perceive that the conserved structural core acts as a supportive framework for residues that form the hydrophobic binding pocket, which are not conserved structural elements. The conserved structural core also acts as a stable infrastructure for other insertions on the ABH fold that are related to the function of the enzyme, such as for the “lids” or “caps” that occur after strand β6 [20, 22–28] and for the insertions at loop_β3→αA_ that we have reviewed in our previous study [9].

Multiple studies refer to the elements of the conserved structural core and their versatile roles in the enzymatic activity of ABH fold proteins. Residue X_IV_ has previously drawn attention because of its position close to the catalytic triad and its interaction with the catalytic residues in several ABH fold enzymes [29–32]. The low rate of amino-acid conservation at position IV hints that residue X_IV_ could affect the enzymatic activity, given that its side chain has a suitable conformation that does not interfere with the catalytic triad, with amino acids of variable side-chain lengths occurring at position IV, including glutamine [33] and tryptophan [34]. Indeed, residue X_IV_ is suggested to be involved in the optimal configuration of the catalytic triad [32, 35] and the enzymatic activity [36–38], to participate in substrate binding [15, 30, 38–41] and to affect the thermostability [35] of ABH fold enzymes. Site-directed mutagenesis of residue X_IV_ in different ABH fold enzymes has confirmed its significance in the enzymatic activity, resulting in a wide range of effects upon its replacement: from decreased [32, 35–37] and acute loss [38] of enzymatic activity, to improved activity [36, 38, 41] and increased thermostability [35]. These observations are consistent with the high rate of amino-acid variability of residue X_IV_.

Residue X_IV+1_ has been suggested to be a determinant of the local geometry [6], frequently occupied by small amino acids (glycine and proline in Table 1) that are suitable for limiting the steric clashes within the active site pocket. Residue X_IV+1_ has also been mentioned as part of the hydrophobic binding pocket [39] and the helical domain that occurs after strand β6 [20], while in some cases it can affect the enzymatic activity [42–44]. To our knowledge, there is only one mutational study that refers to residue X_IV+1_, where its substitution has resulted in nearly complete loss of catalytic activity [45]. The tripeptide residue X_IV_—residue X_IV+1_—residue X_IV+2_ retains its amino-acid conservation in some ABH fold enzyme families, and thus, it is suggested that it can be used as an additional sequence identifier of different ABH fold enzyme families [32, 37]. Residue X_IV+2_ is also extensively studied for the formation of the hydrophobic substrate pocket [22, 25, 32, 37–39, 46–48] and its role in the enzymatic function [32, 49, 50].

Residue X_acid+2_, one of the conserved structural elements that is involved in the coordination of the imidazole ring of the catalytic histidine, is usually a hydrophobic or an aromatic residue located at the entrance of the active site cleft, mostly reviewed for its role in ligand binding [51–56] or the release of products of catalysis [51, 57]. Consequently, the site-directed mutagenesis of residue X_acid+2_ in different ABH enzymes has led to various results, which are consistent with its role in ligand binding, including the compromising [52, 54, 55, 58, 59] or the enhancement [52] of catalytic activity, the alteration of transport tunnels [57, 60], the modification of substrate specificity [53, 55, 56] and the inversion of enantioselectivity [52]. For the remaining residues that coordinate the imidazole ring of the catalytic histidine, including residue X_acid+3_, we have only found a few mutational studies that have resulted in reduced activity [58, 61, 62], with a single study highlighting the residue’s role in the stability of the enzyme and the catalytic activity rather than in ligand binding [58].

### Residue X_IV_ forms conserved interactions with the residues of the catalytic triad in all ABH fold enzymes and thus, can be perceived as the fourth member of a “structural catalytic tetrad”

From a structural point of view, residue X_IV_ undoubtedly is the connecting point of all residues of the acid-base-nucleophile catalytic triad in ABH fold enzymes. Residue X_IV_ is a conserved structural element of the catalytic acid zone that consistently interacts with the catalytic acid when it is located after strand β7, or alternatively, residue X_IV_ is the catalytic acid [8]. Through a contact that is conserved among serine hydrolases, the catalytic histidine also interacts with residue X_IV_ and is speculated to have a functional role in the catalytic mechanism [10]. Here, we have confirmed that residue X_IV_ invariably interacts with the catalytic nucleophile, too. Because of the absolute conservation of the hydrogen-bonding network between residue X_IV_ and the residues of the catalytic triad, we suggest that residue X_IV_ can be viewed as the fourth part of a “structural catalytic tetrad”. This idea is not novel: earlier studies have pointed out residue X_IV_ to be part of the catalytic mechanism in ABH fold enzymes, by providing structural support to the members of the catalytic triad [63–65]. Yet, this observation is not unique in ABH fold enzymes either, because residues that are located near catalytic residues are often studied for their structural role and effects on the catalytic mechanism of enzymes from other families, too [66, 67].

The conserved interaction network between residue X_IV_ and the residues of the catalytic triad is facilitated by the favorable side-chain conformation of residue X_IV_, which does not conflict sterically with the side chains of the catalytic residues under any circumstances. Based on our findings, it is clear that the amino-acid type of residue X_IV_ is not structurally important for the catalytic mechanism itself, but it is the position of residue X_IV_ on the ABH fold that determines its structural role. Thus, we suggest that the position of residue X_IV_ is an equally conserved feature of the catalytic core as those positions that accommodate the catalytic residues. Our claim is further supported when we consider the ABH fold enzymes that have non-canonical catalytic triads [68–73], where the positions of the catalytic residues can be defined more precisely by their spatial arrangement on the catalytic core, rather than their sequence position across the polypeptide chain.

## Conclusions

In this study, we have explored the active sites of enzymes from 40 ABH fold enzyme families, aiming to determine the structural basis for the optimal arrangement of the residues of the acid-base-nucleophile catalytic triad. Complementary to the findings of our previous research, we have here identified a set of conserved structural elements that coordinate the catalytic histidine next to the catalytic nucleophile and the catalytic acid, leading to the fine tuning of the catalytic residues. The results of this study prove that all key units of the catalytic mechanism–the catalytic acid, the catalytic histidine, the catalytic nucleophile and the residues of the oxyanion hole–are coordinated by conserved structural elements that altogether comprise the conserved catalytic core of the ABH fold enzymes.

The relationship between structure and function in ABH fold enzymes is also addressed with this research, because we have demonstrated that the function of the catalytically diverse ABH fold enzymes widely relies on the conserved structural framework that surrounds the catalytic mechanism. Indeed, the conserved structural core secures the structural integrity of the catalytic machinery, while the conserved elements that line the active site of ABH fold enzymes influence the enzymatic activity. In particular, the elements of the conserved structural core participate in ligand binding and are experimentally proven to affect enzymatic properties, such as the substrate specificity, the enantioselectivity, the thermostability and the pH optimum of ABH fold enzymes.

The modification of enzymatic properties is one of the main tasks of protein engineering and the ABH fold enzymes are often selected as targets for tailoring catalysts with improved properties. We expect that our findings can advance the efficacy of protein engineering applications because the conserved structural core offers an ideal template of potential modification sites and thus, we perceive that the conserved elements of the structural core certainly deserve research attention when attempting to customize the catalytic properties of ABH fold enzymes. The usefulness of our results is based on this comprehensive analysis of the active site of ABH fold enzymes that takes into consideration the conserved structural features of the ABH fold common among all ABH fold enzymes, while taking less notice of the individual variations that occur among the different classes of catalysts that belong to the ABH fold structural family.

Finally, the identification of the conserved structural core signifies that the function of all ABH fold enzymes is based on a minimal, but stabile infrastructure, which could explain the widespread occurrence of ABH fold enzymes in nature and their ability to catalyze various reactions on different substrates. However, the alpha/beta-Hydrolases are not the only enzymes with an acid-base-nucleophile catalytic triad and subsequently, the notion of the conserved structural core around the catalytic mechanism should be explored in other classes of enzymes that use an acid-base-nucleophile catalytic triad in order to catalyze reactions (such as trypsins, chymotrypsins, subtilisins and serine proteases) or in other enzymes that have alpha/beta-hydrolases fold structures.

P.S. One of the manuscript reviewers asked us an important question: “whether the weak hydrogen-bonding interactions (3.8 Å and more) do really contribute to protein stability at ambient temperatures?” To answer this question, we analyzed the X-ray diffraction data of three proteins obtained at cryogenic and room temperatures. The analysis results are presented in S1C Table. Two conclusions follow from them:

1. BIOVIA (Accelrys) Discovery Studio [74] enables the reliable inclusion of calculated hydrogen atoms in enzyme structures with resolution ≤ 2.2 Å.
2. The conformational characteristics of weak hydrogen bonds do not change significantly when enzyme structures are compared at cryogenic and room temperatures.

## Materials and methods

The representative structures of our dataset are selected based on the categorization of ABH fold families that is provided by the Structural Classification of Proteins (SCOP) database [11]. The SCOP database classifies 41 ABH families, which altogether contain 128 different entries of ABH proteins. We have excluded the single entry from the TTHA1544-like family (SCOP Family #39) because it is not a hydrolase [75]. The remaining 40 ABH enzyme families contain in total 127 entries, from which we have chosen the highest resolution structure to represent each one of the 40 ABH fold enzyme families. Additionally, we have included the two carboxylestarases SshEstI and EstFa_R from the study of Ohara *et al*. [13] that we use as reference structures for our analysis. All protein structures are obtained from Protein Data Bank (PDB) [12].

The results of this study are presented in two tables, with each table containing a different number of entries. Table 1 contains representative structures from the 40 ABH fold enzymes families plus the carboxylesterase SshEstI (second row); all structures are unligated, except for SshEstI. The conserved interactions that are shown in Table 1 refer to the two contacts that coordinate the catalytic nucleophile-histidine pair (column CA/Nucleophile–O/X_IV_ and column O/Nucleophile–CA/X_IV+1_) and the additional conserved interaction that is located at the catalytic site of ABH fold enzymes (Sc/(Nucleophile+4)–O/X_IV+2_).

Table 2 contains ligand-bound structures from 28 ABH fold enzyme families plus the carboxylesterase SshEstI (second row), while three of the 28 ABH fold families are represented by more than one structures (SCOP families: #12 Haloalkane dehalogenase, #15 Biotin biosynthesis protein BioH, #18 Epoxide hydrolase). The ligand-bound structures of Table 2 correspond to the ligand-free structures of Table 1, and thus, in Table 2 we have omitted the twelve ABH fold families for which we have found no equivalent ligand-bound structures. The columns of Table 2 describe the residues of the conserved structural core that are involved in ligand binding, whereby the residues are distributed in the columns based on their position close to the one of the key units of the catalytic mechanism.

For all structural analyses, such as identification of hydrogen bonds, hydrophobic or other types of weak interactions, we have used the BIOVIA (Accelrys) Discovery Studio [74] (http://accelrys.com/products/collaborativescience/bioviadiscoverystudio/), the Ligand-Protein Contacts software and the Contacts of Structural Units software (LPC; CSU) [76]. We have identified the weak hydrogen bonds from CH–O contacts in structures with at least 2.0 Å resolution based on geometrical criteria given in Derewenda *et al*. [77] and with distances C …O ≤ 4.1 Å and H …O ≤ 3.0 Å, and the weak hydrogen bonds from CH–π contacts with distance between 3.4 Å and 6Å [78, 79].

Lastly, for the visualization and analysis of the structural data, we have used BIOVIA (Accelrys) Discovery Studio [74] and Bodil [80]. Figures are drawn with MolScript [81].

## Supporting information

S1 Table Interactions within the catalytic core in ABH fold enzyme families. (DOCX)
