# Supplementary material for: The acid-base-nucleophile catalytic triad in ABH-fold enzymes is coordinated by a set of structural elements
Source: PLoS One. 2020 Feb 21;15(2):e0229376. doi: 10.1371/journal.pone.0229376 (PMC7034887; doi:10.1371/journal.pone.0229376)
Supplement: S1 Table — (DOCX) [file pone.0229376.s001.docx]

**Supplementary information for “The acid-base-nucleophile catalytic triad in ABH-fold enzymes is coordinated by a set of structural elements.”**

Alexander Denesyuk,^1,2^ Polytimi S. Dimitriou,^1^ Mark S. Johnson,^1^ Toru Nakayama^3^ and Konstantin Denessiouk^1^

^1^Structural Bioinformatics Laboratory, Biochemistry, Faculty of Science and Engineering, Åbo Akademi University, Turku 20520, Finland

^2^Institute for Biological Instrumentation of the Russian Academy of Sciences, Federal Research Center “Pushchino Scientific Center for Biological Research of the Russian Academy of Sciences”, Pushchino 142290, Russia

^3^Department of Biomolecular Engineering, Graduate School of Engineering, Tohoku University, Sendai, Miyagi 980-8579, Japan

**S1 Table. Interactions within the catalytic core in ABH fold enzyme families.**

**S1A Table. Inventory of interactions between the catalytic acid and the catalytic histidine in 40 ABH fold enzyme families.** The catalytic acid forms one weak hydrogen bond with the CA atom of the catalytic histidine (CA/Base – (OD, OE)/Acid) by using one of its side-chain oxygen atoms and a second weak hydrogen bond with the CB atom of the catalytic histidine (CB/Base – (OD, OE)/Acid) by using the other side-chain oxygen atom. The interactions from the carboxylesterase SshEstI [1] (SCOP [2] family #2, PDB ID:3WJ1_A) are shown in the first entry of the table, and correspond to the interactions illustrated in Figs 2 and 3. SCOP family #12 is represented by two structures in order to properly reflect the local structural variations in ABH fold enzymes that have their catalytic acid at the canonical position (at the turn that follows strand β7, e.g. in structure PDB ID:1B6G_A) or at its alternate position (at the position of residue X_IV_, e.g. in structure PDB ID:1MJ5_A). “None” indicates the absence of a contact (for example, in SCOP family #38). Values in parentheses correspond to the distance of the hydrogen bond to the hydrogen atom.

| SCOP family # / SCOP family name / Reference | PDB ID | CA/Base – (OD, OE)/Acid | CB/Base – (OD, OE)/Acid |
| --- | --- | --- | --- |
| 2. Carboxylesterase [1] | 3WJ1_A | CA/H274-OD2/D244 3.7 (3.0) | CB/H274-OD1/D244 3.5 (2.5) |
| 1. Acetylcholinesterase-like [3] | 1QE3_A | CA/H399-OE1/E310 3.3 (2.5) | CB/H399-OE1/E310 3.3 (2.9) |
| 2. Carboxylesterase [4] | 1LZL_A | CA/H290-OD2/D260 3.8 (3.1) | CB/H290-OD1/D260 3.5 (2.6) |
| 3. Mycobacterial antigens [5] | 1DQZ_A | CA/H260-OE1/E228 3.4 (2.8) | CB/H260-OE1/E228 3.4 (2.9) |
| 4. Hypothetical protein TT1662 [6] | 1UFO_A | CA/H217-OD1/D183 3.6 (2.8) | CB/H217-OD2/D183 3.2 (2.2) |
| 5. PepX catalytic domain-like [7] | 3PUI_A | CA/H287-OD2/D259 3.4 (2.8) | CB/H287-OD2/D259 3.4 (3.1) |
| 6. Prolyl oligopeptidase, C-terminal domain [8] | 1H2W_A | CA/H680-OD2/D641 3.5 (2.6) | CB/H680-OD1/D641 3.3 (2.6) |
| 7. DPP6 catalytic domain-like [9] | 1ORV_A | CA/H740-OD2/D708 3.3 (2.3) | CB/H740-OD1/D708 3.2 (2.4) |
| 8. Serine carboxypeptidase-like [10] | 3SC2_A, B | CA/H397-OD2/D338 4.1 (3.3) | CB/H397-OD1/D338 3.2 (2.3) |
| 9. Gastric lipase [11] | 1HLG_A | CA/H353-OD2/D324 3.0 (2.1) | CB/H353-OD1/D324 3.2 (2.4) |
| 10. Proline aminopeptidase-like [12] | 1MTZ_A | CA/H271-OD1/D244 3.7 (2.6) | CB/H271-OD2/D244 3.4 (2.9) |
| 11. Acetyl xylan esterase-like [13] | 1L7A_A | CA/H298-OD2/D269 3.4 (2.8) | CB/H298-OD1/D269 3.3 (2.4) |
| 12. Haloalkane dehalogenase [14], [15] | 1B6G_A | CA/H289-OD2/D260 3.5 (2.6) | CB/H289-OD1/D260 3.2 (2.3) |
|  | 1MJ5_A | CA/H272-OE1/E132 3.7 (2.8) | CB/H272-OE1/E132 3.9 (3.7) |
| 13. Dienelactone hydrolase [16] | 1ZI9_A | CA/H202-OD2/D171 3.7 (3.1) | CB/H202-OD1/D171 3.7 (2.7) |
| 14. Carbon-carbon bond hydrolase [17] | 2OG1_A | CA/H265-OD2/D237 3.5 (2.5) | CB/H265-OD1/D237 3.4 (2.5) |
| 15. Biotin biosynthesis protein BioH [18] | 4ETW_A | CA/H235-OD2/D207 3.5 (2.5) | CB/H235-OD1/D207 3.6 (2.9) |
| 16. Aclacinomycin methylesterase RdmC [19] | 1Q0R_A | CA/H276-OD2/D248 4.9 (4.3) | CB/H276-OD1/D248 3.7 (2.8) |
| 17. Carboxylesterase/lipase [20] | 4DIU_A | CA/H222-OD2/D192 3.5 (2.6) | CB/H222-OD1/D192 3.4 (2.6) |
| 18. Epoxide hydrolase [21] | 1QO7_A | CA/H374-OD1/D348 3.5 (2.6) | CB/H374-OD1/D348 3.5 (3.0) |
| 19. Haloperoxidase [22] | 1BRT_A | CA/H257-OD2/D228 3.4 (2.5) | CB/H257-OD1/D228 3.3 (2.4) |
| 20. Thioesterases [23] | 1EI9_A | CA/H289-OD2/D233 3.7 (2.9) | CB/H289-OD1/D233 3.6 (2.6) |
| 21. Carboxylesterase/thioesterase 1 [24] | 1FJ2_A | CA/H203-OD2/D169 3.4 (2.5) | CB/H203-OD1/D169 3.3 (2.4) |
| 22. Ccg1/TafII250-interacting factor B (Cib) [25] | 1IMJ_A | CA/H188-OD2/D162 3.4 (2.4) | CB/H188-OD1/D162 3.9 (3.2) |
| 23. A novel bacterial esterase [26] | 1QLW_A | CA/H298-OE1/E230 3.3 (2.3) | CB/H298-OE1/E230 3.5 (3.2) |
| 24. Lipase [27] | 1JFR_A | CA/H209-OD2/D177 3.7 (2.8) | CB/H209-OD1/D177 3.4 (2.5) |
| 25. Fungal lipases [28] | 1TCA_A | CA/H224-OD2/D187 3.8 (2.9) | CB/H224-OD1/D187 3.4 (2.6) |
| 26. Bacterial lipase [29] | 1ISP_A | CA/H156-OD2/D133 3.7 (2.9) | CB/H156-OD1/D133 3.4 (2.5) |
| 27. Pancreatic lipase, N-terminal domain [30] | 1BU8_A | CA/H263-OD2/D176 3.5 (2.5) | CB/H263-OD2/D176 4.0 (4.2) |
| 28. Hydroxynitrile lyase-like [31] | 3C6X_A | CA/H235-OD2/D207 3.7 (2.9) | CB/H235-OD1/D207 3.5 (2.7) |
| 29. Thioesterase domain of polypeptide, polyketide and  fatty acid synthases [32] | 1JMK_C | CA/H207-OD2/D107 3.7 (3.0) | CB/H207-OD2/D107 3.4 (2.7) |
| 30. Cutinase-like [33] | 1BS9_A | CA/H187-OD2/D175 3.3 (2.4) | CB/H187-OD2/D175 3.4 (3.1) |
| 31. YdeN-like [34] | 1UXO_A | CA/H164-OD2/D137 3.5 (2.6) | CB/H164-OD1/D137 3.5 (2.7) |
| 32. Putative serine hydrolase Ydr428c [35] | 1VKH_A | CA/H243-OD2/D211 3.8 (2.9) | CB/H243-OD1/D211 3.4 (2.5) |
| 33. Acylaminoacid-releasing enzyme,  C-terminal domain [36] | 1VE6_A | CA/H556-OD2/D524 3.7 (3.1) | CB/H556-OD1/D524 3.4 (2.5) |
| 34. Hypothetical esterase YJL068C [37] | 1PV1_A | CA/H276-OD2/D241 3.6 (3.0) | CB/H276-OD1/D241 3.2 (2.2) |
| 35. Hypothetical protein VC1974 [38] | 1R3D_A | CA/H240-OD2/D218 3.3 (2.5) | CB/H240-OD2/D218 3.3 (3.0) |
| 36. Atu1826-like [39] | 2I3D_A | CA/H190-OD2/D157 3.4 (2.5) | CB/H190-OD1/D157 3.3 (2.4) |
| 37. PHB depolymerase-like [40] | 2D80_A | CA/H155-OD1/D121 3.5 (2.7) | CB/H155-OD2/D121 3.4 (2.4) |
| 38. IroE-like [41] | 2GZR_A | None | None |
| 40. O-acetyltransferase [42] | 2B61_A | CA/H337-OD2/D304 3.5 (2.6) | CB/H337-OD1/D304 3.3 (2.4) |
| 41. 2,6-dihydropseudo-oxynicotine hydrolase-like [43] | 2JBW_A | CA/H329-OD2/D300 3.5 (2.4) | CB/H329-OD1/D300 3.5 (2.8) |

**S1B Table. Inventory of interactions that occur within the local structure that surrounds the catalytic acid zone in 40 ABH fold enzyme families.** The coordination of the catalytic histidine involves hydrophobic and CH–π interactions (set I) that are formed between the imidazole ring and residues that are located two or three sequence positions after the catalytic acid or other neighboring residues. The residues that follow the catalytic acid have an elaborate structural role, because they often interact with residues at the C-terminus of strand β6 (set II) and they are part of the structural Asx-motif [44] (set III), which also interacts with the catalytic acid zone (set IV). The representative structure of ABH fold enzyme family IroE-like (SCOP family #38) does not have any of the mentioned interactions (indicated as “None”), because the corresponding protein is suggested to use a base-nucleophile catalytic dyad to catalyze reactions [41]. The first entry lists the interactions of the carboxylesterase SshEstI (SCOP family #2, PDB ID:3WJ1_A), corresponding to those (set I and set II) illustrated in Fig 3. In four ABH fold families [SCOP families #12 (PDB ID:1MJ5_A), #23, #27 and #29], residue X_IV_ is the catalytic acid residue. SCOP family #12 is represented by two structures in order to properly reflect the local structural variations in ABH fold enzymes where the catalytic acid is located at the canonical position (at the turn that follows strand β7, e.g. in structure PDB ID:1B6G_A) or at its alternate position (at the position of residue X_IV_, e.g.in structure PDB ID:1MJ5_A). Residues highlighted with green color signify that the Asx-motif is formed by the catalytic acid and the residue located two positions after the catalytic acid; residues highlighted with blue signify that the Asx-motif is formed by the catalytic acid and the two residues located two and three positions after the catalytic acid.

| SCOP family # /  SCOP family name | PDB ID  Reference | Set I | Set II | Set III | Set IV |
| --- | --- | --- | --- | --- | --- |
| 2. Carboxylesterase | 3WJ1_A  [1] | CD2/L198-π/H274 3.5 (2.6)  CD1/L246-CE1/H274 3.5 | CD1/L246-O/Y177 3.9 (3.1)  CB/L246-O/P178 3.8 (2.9) | OD1/D244-N/L246 2.9  OD1/D244-CB/L246 3.5 (2.8) | O/R247-CG2/T240 3.8 (2.9)  CD/R247-O/A241 3.4 (2.8)  NH1/R247-O/E242 2.9  N/R247-O/D244 3.0 |
| 1. Acetylcholin-esterase-like | 1QE3_A  [3] | CZ/F314-π/H399 4.0 (3.2)  CE2/F363-CE1/H399 3.3 | CD2/F363-O/S215 3.3 (2.6)  CE2/F363-O/S215 3.3 (2.6) | O/E310-CD1/F314 3.9 (3.1)  E310-F363 stacking | CD1/W364-OG1/T306 4.1 (3.1)  CG2/T359-O/T307 4.4 (3.6)  CG2/T359-O/R308 3.7 (2.8)  N/L313-O/E310 3.2 |
| 2. Carboxylesterase | 1LZL_A  [4] | CB/W209-π/H290 3.9 (3.1)  CD1/L262-CE1/H290 3.4 | CD1/L262-O/I188 3.5 (2.9)  CB/L262-O/P189 3.7 (2.7) | OD1/D260-N/L262 2.9  OD1/D260-CB/L262 3.4 (2.7) | O/R263-CG2/T256 3.6 (2.7)  CB/R263-O/M257 3.3 (2.4)  NH1/R263-O/E258 2.8  N/R263-O/D260 2,9 |
| 3. Mycobacterial antigens | 1DQZ_A  [5] | CB/L227-π/H260 4.1 (3.3)  CG2/T231-CE1/H260 5.0 | CG2/T231-O/S148 4.2 (3.2)  OG1/T231-O/G149 2.8 | O/L227-N/T231 3.1  O/L227-CG2/T231 3.6 (2.7)  O/E228-CB/L232 3.5 (2.8) | CA/L232-SG/C209 3.6  CB/L232-O/G210 3.8 (2.8)  CA/G229-O/N211 4.3 (3.6)  N/L232-O/E228 3.1 |
| 4. Hypothetical protein TT1662 | 1UFO_A  [6] | CB/I185-π/H217 3.8 (2.9)  CG2/V186-CE1/H217 3.7 | CG2/V186-O/I136 3.8 (3.0)  CG1/V186-O/G137 3.8 (2.8) | OD2/D183-N/I185 2.9  OD2/D183-N/V186 2.9  OD1/D183-CB/V186 3.9 (2.9) | CD1/L188-CD2/H179 4.0  CB/L188-O/G180 4.7 (3.7)  CB/L188-O/S181 4.3 (3.4)  CA/P187-O/D183 3.5 (2.4) |
| 5. PepX catalytic domain-like | 3PUI_A  [7] | CD2/L407- π/H287 4.1 (3.3)  CE1/F261-NE2/H287 3.5 | CB/F261-O/M141 3.5 (3.2)  CB/F261-O/A142 3.7 (2.6) | OD1/D259-N/F261 3.0  OD1/D259-CB/F261 3.2 (2.4) | CG2/V262-CB/A255 4.3  CB/V262-O/G256 3.2 (2.3)  CG1/V262-O/W257 4.1 (3.1)  N/V262-O/D259 3.0 |
| 6. Prolyl oligopeptidase,  C-terminal domain | 1H2W_A  [8] | CB/R643-π/H680 3.8 (3.0)  CG2/V644-CE1/H680 3.4 | CG2/V644-O/V578 3.8 (3.0)  CG1/V644-O/G579 3.5 (2.8) | OD1/D641-N/R643 3.0  OD1/D641-N/V644 3.0  OD2/D641-CB/V644 4.1 (3.1) | CA/P646-OG1/T637 3.8 (2.9)  CG/P646-O/A638 3.1 (2.3)  CG/P646-O/D639 4.5 (3.6)  CA/V645-O/D641 3.3 (2.4) |
| 7. DPP6 catalytic domain-like | 1ORV_A  [9] | CB/N710-π/H740 4.3 (3.3)  CG2/V711-CE1/H740 3.4 | CG2/V711-O/A654 4.0 (3.2)  CG1/V711-O/P655 3.1 (2.4) | OD1/D708-N/N710 2.9  OD1/D708-N/V711 2.9  OD2/D708-CB/V711 4.1 (3.1) | CA/F713-π/H704 3.9 (2.8)  CD1/F713-O/G705 3.8 (2.9)  CB/F713-O/T706 4.1 (3.2)  CA/H712-O/D708 3.6 (2.5) |
| 8. Serine carboxypeptidase-like | 3SC2_A, B [10] | CD1/Y239-π/H397 3.7 (2.8)  CG2/V341-CE1/H397 4.1 | CG2/V341-O/N176 3.7 (3.1)  CG1/V341-O/G177 3.6 (2.8) | OD1/D338-N/V340 3.1  OD2/D338-N/V341 3.0  OD2/D338-CB/V341 4.0 (3.3) | CA/L343-OG/S334 3.4 (2.5)  CB/L343-O/G335 5.0 (4.0)  CD2/L343-CB/D336 3.9  CB/P342-O/D338 3.8 (2.9) |
| 9. Gastric lipase | 1HLG_A [11] | CB/L326-π/H353 4.1 (3.3)  CB/A327-CE1/H353 3.9 | CB/A327-O/A180 3.7 (3.1)  CB/A327-O/P181 3.3 (2.6) | OD1/D324-N/L326 2.9  OD1/D324-N/A327 2.6  OD2/D324-CB/A327 3.4 (2.3) | CA/P329-ND2/N320 4.5  CG/P329-O/G321 3.5 (2.6)  CG/P329-O/G322 3.8 (2.9)  CA/D328-O/D324 3.2 (2.3) |
| 10. Proline aminopeptidase-like | 1MTZ_A [12] | OE2/E245-π/H271 3.8  CG2/V246-CE1/H271 4.0 | CG2/V246-O/G129 3.6 (2.8)  CG1/V246-O/G130 3.3 (2.7) | OD2/D244-N/V246 3.0  OD2/D244-CB/V246 3.7 (3.0) | CA/P248-CG2/V240 3.9  CD/P248-O/G241 3.8 (2.8)  CD/P248-O/E242 3.9 (3.3)  CA/T247-O/D244 3.8 (3.0) |
| 11. Acetyl xylan esterase-like | 1L7A_A [13] | CB/V271-π/H298 3.7 (2.9)  OG1/T272-ND1/H298 3.0 | OG1/T272-O/Y204 3.6  CG2/T272-O/Y204 3.6 (2.7)  CG2/T272-O/P205 3.8 (3.3) | OD1/D269-N/V271 2.8  OD1/D269-N/T272 2.9  OD2/D269-OG1/T272 2.8 | CA/P274-CG1/I265 3.8  CG/P274-O/G266 3.6 (2.6)  CG/P274-O/L267 3.6 (2.8)  CA/P273-O/D269 3.4 (2.3) |
| 12. Haloalkane dehalogenase | 1B6G_A [14] | CD2/L262-π/H289 4.4 (3.3)  CD1/L263-CE1/H289 3.9 | CD1/L263-O/N148 3.7 (2.9)  SD/M268-O/A149 3.4 | OD1/D260-N/L262 2.9  OD2/D260-N/L263 3.0  OD2/D260-CB/L263 3.6 (2.8) | CD/P265-CG2/I256 4.0  CA/G264-O/G257 4.4 (3.6)  CD/P265-O/M258 4.6 (4.0)  CA/G264-O/D260 4.0 (3.2) |
| 12. Haloalkane dehalogenase | 1MJ5_A [15] | CB/L177-CB/H272 3.8  CD2/L248-CE1/H272 4.6 | CD2/L248-O/E132 4.3 (3.4)  CD2/L248-O/A133 3.3 (2.8) | OE1/E132-N/L248 2.8  OE1/E132-CB/L248 3.5 (2.9)  CG/E132-O/A271 3.6 (2.9) | O/T249-OD1/N242 3.8 |
| 13. Dienelactone hydrolase | 1ZI9_A  [16] | CE2/F173-π/H202 3.7 (3.0)  CB/V174-CD2/H202, 3.6 | CG2/V174-O/G146 4.6 (4.2) | OD1/D171-N/F173 2.9  OD1/D171-CB/F173 3.4 (2.5) | CG2/V174-CE/M167 3.9  CB/V174-O/G168 5.1 (4.0)  N/F173-O/D171 2.8 |
| 14. Carbon-carbon bond hydrolase | 2OG1_A [17] | CD2/F239-π/H265 3.7 (2.8)  CG2/V240-CE1/H265 3.6 | CG2/V240-O/G136 3.4 (2.4)  CG1/V240-O/P137 4.0 (3.2) | OD1/D237-N/F239 2.7  OD1/D237-N/V240 3.0  OD2/D237-CG2/V240 3.2 (2.7) | O/V240-NE1/W233 3.1  CD1/L242-O/G234 4.6 (3.5)  CD2/L242-O/R235 4.4 (3.3)  O/V240-O/D237 3.8 |
| 15. Biotin biosynthesis protein BioH | 4ETW_A [18] | CD1/L209-π/H235 3.7 (2.7)  CG2/V210-CE1/H235 4.4 | CG2/V210-O/A106 3.6 (2.7)  CG1/V210-O/S107 3.9 (3.0) | OD1/D207-N/L209 2.8  OD1/D207-N/V210 3.5  OD2/D207-CB/V210 3.8 (2.9) | CG2/V215-π/Y203 3.4 (2.4)  CB/R212-O/G204 4.8 (3.8)  NH1/R212-O/Y205 3.1  CA/P211-O/D207 4.0 (3.1) |
| 16. Aclacinomycin methylesterase RdmC | 1Q0R_A [19] | CD1/I250-π/H276 3.5 (2.5) | CB/A251-O/L126 4.2 (3.5)  CB/A251-O/G127 3.5 (2.8) | OD1/D248-N/I250 3.0  OD1/D248-N/A251 3.1  OD2/D248-CB/A251 4.1 (3.1) | O/A251-OE1/Q244 3.2  CB/A253-O/E246 5.0 (4.4)  CA/P252-O/D248 3.3 (2.6) |
| 17. Carboxylesterase/  lipase | 4DIU_A [20] | CB/M194-π/H222 3.9 (3.0)  CG2/I195-CE1/H222 3.5 | CD1/I195-O/C115 3.7 (2.9)  CD1/I195-O/A116 3.5 (3.2) | OD1/D192-N/M194 2.7  OD1/D192-N/I195 2.8  OD2/D192-CG1/I195 3.5 (2.5) | CD1/I195-OE1/Q188 3.8 (2.7)  CB/P197-O/A189 3.7 (2.7)  CG/P197-O/R190 3.4 (2.6)  O/L195-O/D192 3.5 |
| 18. Epoxide hydrolase | 1QO7_A [21] | CD1/L349-π/H374 4.1 (3.4)  CE3/W284-CD2/H374, 3.6 | OD2/D348-O/N214 3.0 | OD1/D348-N/L349 2.5  OD1/D348-CB/L349 3.5 (2.5) | O/V352-CE1/F344 3.4 (2.6)  CB/P351-O/F345 4.7 (3.7)  CB/P351-O/P346 3.9 (3.1)  CD/P351-O/D348 3.4 (2.4) |
| 19. Haloperoxidase | 1BRT_A [22] | CG2/T230-π/H257 4.1 (3.6)  CD1/L231-CE1/H257 3.6 | CD1/L231-O/A123 3.5 (2.5)  CB/L231-O/S124 4.1 (3.3) | OD1/D228-N/T230 2.8  OD1/D228-N/L231 2.8  OD2/D228-CB/L231 4.3 (3.3) | O/L231-NE2/H224 2.8  CB/L233-O/G225 3.9 (2.9)  CG2/L233-O/T226 4.4 (3.9)  CA/P232-O/D228 3.6 (2.6) |
| 20. Thioesterases | 1EI9_A  [23] | CB/I235-π/H289 4.7 (3.8)  CG2/V236-π/H289 4.2 3.4  CG2/V236-CE1/H289 3.7 | CG2/V236-O/G140 4.5 (3.5)  CA/E242-O/G141 3.1 (2.0) | OD1/D233-N/I235 3.0  OD1/D233-N/V236 2.9  OD2/D233-CB/V236 4.1 (3.2) | O/V239-CD/K229 3.4 (2.7)  CB/V239-O/F230 4.7 (3.8)  CG1/V239-O/L231 5.2 (4.2)  CG1/V239-O/D233 3.5 (3.0) |
| 21. Carboxylesterase/  thioesterase 1 | 1FJ2_A  [24] | CD1/L171-π/H203 4.0 (3.5)  CG2/V172-CE1/H203 3.6 | CG2/V172-O/S138 4.1 (3.3)  CG1/V172-O/C139 4.1 (3.5) | OD1/D169-N/L171 2.8  OD1/D169-N/V172 2.8  OD2/D169-CB/V172 4.0 (2.9) | O/V172-NE2/H165 2.9  CB/L174-O/G166 4.0 (3.0)  CA/P173-O/D169 4.0 (3.1) |
| 22. Ccg1/TafII250-interacting factor B (Cib) | 1IMJ_A  [25] | CE/M164-π/H188 3.9 (3.0)  CB/M164-CE1/H188 4.3 | CB/M164-O/A135 4.9 (4.0)  SD/M164-O/A135 4.6 | OD1/D162-N/M164 2.9  OD2/D162-CB/M164 3.9 (2.9) | CA/G165-π/Y158 3.9 (2.5)  CA/G165-O/G159 4.8 (3.8)  N/G165-O/D162 2.9 |
| 23. A novel bacterial esterase | 1QLW_A [26] | CZ2/W262-π/H298 3.6 (3.1)  CD1/F145-CD2/H298 3.6 | CH2/W262-O/E230 3.5 (2.7)  CZ2/W262-O/P231 3.9 (2.9) | OE1/E230-π/W262 4.1 | CD1/L266-π/F252 4.0 (3.2) |
| 24. Lipase | 1JFR_A  [27] | CB/V179-π/H209 3.6 (2.7)  CB/A180-CE1/H209 3.9 | CB/A180-O/T154 4.0 (3.3)  CB/A180-O/G155 3.4 (2.5) | OD1/D177-N/V179 2.7  OD1/D177-N/A180 2.7  OD2/D177-CB/A180 4.0 (3.0) | CG1/V182-CA/G173 3.8  CG1/V182-O/A174 3.6 (2.8)  CG2/V182-O/D175 5.2 (4.3)  CA/P181-O/D177 3.5 (2.5) |
| 25. Fungal lipases | 1TCA_A [28] | CB/I189-π/H224 3.9 (3.0)  CG2/V190-CE1/H224 3.7 | CG2/V190-O/A132 4.3 (3.3)  CG1/V190-O/P133 4.5 (3.4) | OD1/D187-N/I189 2.9  OD1/D187-N/V190 2.9  OD2/D187-CB/V190 4.0 (3.1) | O/Q193-CE2/Y183 3.5 (2.6)  CB/Q193-O/S184 3.5 (3.0)  NE2/Q191-O/A185 3.0  NE2/Q191-O/D187 2.8 |
| 26. Bacterial lipase | 1ISP_A  [29] | CB/I135-π/H156 4.0 (3.1)  CG2/V136-CE1/H156 3.4 | CG2/V136-O/G103 3.6 (2.7)  CG1/V136-O/G104 3.6 (2.7) | OD1/D133-N/I135 2.8  OD1/D133-N/V136 2.9  OD2/D133-CB/V136 4.3 (3.3) | CG/N138-CD1/Y129 3.4  ND2/N138-O/S130 3.0  OD1/N138-CA/S131 3.5 (2.5)  CA/M137-O/D133 4.9 (3.9) |
| 27. Pancreatic lipase,  N-terminal domain | 1BU8_A [30] | CB/F215-π/H263 3.9 (2.9)  CD1/F215-CE1/H263 4.1 | CA/G216-O/D176 4.4 (3.6)  CA/G216-O/P177 3.4 (2.5) | OD2/D176-CA/G216 3.5 (2.7) | O/M217-ND1/H203 2.8  CB/S218-O/S206 3.4 (2.3)  CA/S218-CB/S206 3.6 |
| 28. Hydroxynitrile lyase-like | 3C6X_A [31] | CG2/I209-π/H235 3.7 (3.3)  CD1/F210-CE1/H235 4.0 | CD2/F210-O/N104 3.3 (3.2)  CE2/F210-O/S105 4.5 (3.7) | OD1/D207-N/I209 2.9  OD1/D207-N/F210 2.9  OD2/D207-CB/F210 3.5 (2.5) | OE1/Q215-CB/W203 3.2 (2.7)  NE2/Q215-O/T204 3.9  CG/P212-O/D205 3.4 (2.6)  CA/L211-O/D207 3.8 (2.9) |
| 29. Thioesterase domain of polypep-tide, polyketide and fatty acid synthases | 1JMK_C [32] | O/D107-ND1/H207 3.1  OD2/D125-CD2/H207 3.1 (2.9) | F181-Y109 stacking | None | OH/Y109-CG2/T176 3.8 (3.0) |
| 30. Cutinase-like | 1BS9_A  [33] | CD2/Y177-π/H187 3.7 (2.8)  CB/Y177-CE1/H187 3.9 | CB/C178-O/G132 3.7 (2.6)  CB/C178-O/D133 3.2 (2.5) | OD1/D175-N/Y177 2.8  OD1/D175-N/C178 3.1  OD2/D175-SG/C178 3.2 | SG/C178-SG/C171 2.0  CB/C179-O/D172 3.3 (2.7)  N/G181-O/A173 3.4  N/C179-O/D175 3.3 |
| 31. YdeN-like | 1UXO_A [34] | CB/I139-π/H164 4.1 (3.2)  CG2/V140-CE1/H164 3.6 | CG2/V140-O/S97 3.6 (2.6)  CG1/V140-O/G98 3.9 (3.1) | OD1/D137-N/I139 2.8  OD1/D137-N/V140 3.0  OD2/D137-CB/V140 4.0 (3.0) | CD1/F142-CB/A133 3.7  CB/F142-O/S134 3.7 (2.6)  CB/F142-O/K135 3.9 (3.1)  CA/P141-O/D137 4.0 (3.0) |
| 32. Putative serine hydrolase Ydr428c | 1VKH_A [35] | CB/L213-π/H243 4.5 (3.5)  CD1/L214-CE1/H243 3.9 | CD1/L214-O/D151 3.6 (2.9)  CD1/L214-O/G152 3.6 (2.9) | OD1/D211-N/L213 2.8  OD1/D211-N/L214 2.7  OD2/D211-CB/L214 3.9 (2.9) | O/L214-NE2/H207 2.8  O/L214-O/S208 4.9  O/S214-O/D211 3.5 |
| 33. Acylamino-acid-  releasing enzyme,  C-terminal domain | 1VE6_A [36] | CD/R526-π/H556 4.0 (2.9)  OG1/T527-CE1/H556 3.4 (2.9) | OG1/T527-O/A469 3.1  CB/T527-O/S470 3.7 (2.6) | OD1/D524-N/R526 2.9  OD1/D524-N/T527 2.9  OD2/D524-OG1/T527 2.9 | O/T527-NE2/H520 2.7  CB/L529-O/P521 4.4 (3.3)  CB/L529-O/Q522 3.9 (3.4)  CA/P528-O/D524 3.5 (2.5) |
| 34. Hypothetical esterase YJL068C | 1PV1_A [37] | CD1/F243-π/H276 3.9 (3.3)  CD1/L248-CE1/H276 4.2 | CD1/L248-O/A187 3.8 (2.7)  CB/L248-O/P188 3.3 (2.5) | OD1/D241-N/F243 3.1  O/F243-N/H247 2.9  O/F243-N/L248 3.4  OD2/D241-CD1/L248 3.3 (2.7) | CD1/L244-CG2/V237 3.5  CD2/L244-O/G238 4.2 (3.1)  CD2/L244-O/D239 4.1 (3.5)  N/L244-O/D241 2.9 |
| 35. Hypothetical protein VC1974 | 1R3D_A [38] | CB/V160-π/H240 4.1 (3.3)  π/F221-CE1/H240 3.5 (2.6) | CE2/F221-O/G118 3.4 (3.2)  CE2/F221-O/G119 4.2 (3.4) | OD1/D218-N/K220 3.6  OD1/D218-N/F221 3.3  OD2/D218-CB/F221 3.4 (2.4) | CA/Q222-SG/C214 3.8  CB/Q222-O/G215 4.1 (3.0)  CG/Q222-O/E216 4.6 (3.6)  N/Q222-O/D218 2.9 |
| 36. Atu1826-like | 2I3D_A  [39] | CG1/V159-π/H190 3.7 (3.1)  CB/A160-CE1/H190 4.3 | CB/A160-O/A131 4.8 (4.3)  CB/A160-O/P132 3.2 (2.6) | OD1/D157-N/V159 2.8  OD1/D157-N/A160 2.8  OD2/D157-CB/A160 4.0 (3.0) | CA/E162-ND2/N153 4.2  CG/E162-O/G154 4.3 (3.7)  OE1/E162-O/D155 4.0  CA/P161-O/D157 3.4 (2.4) |
| 37. PHB depolymerase-like | 2D80_A  [40] | CG2/T123-π/H155 4.0 (3.5)  CG2/V124-CE1/H155 3.5 | CG2/V124-O/A64 3.4 (2.4)  CG1/V124-O/G65 3.2 (2.5) | OD2/D121-N/T123 2.8  OD2/D121-N/V124 3.0  OD1/D121-CB/V124 4.3 (3.4) | CG/P126-OG1/T117 4.0 (3.2)  CG/P126-O/G118 3.6 (2.8)  CG/P126-O/S119 3.8 (3.0)  CA/G125-O/D121 3.4 (2.5) |
| 38. IroE-like | 2GZR_A [41] | None | None | None | None |
| 40. O-acetyl-transferase | 2B61_A  [42] | CD2/L306-π/H337 4.3 (3.2)  CD2/F307-CE1/H337 3.1 | CB/F307-O/C167 4.0 (3.0) | OD1/D304-N/L306 2.8  OD1/D304-N/F307 2.8  OD2/D304-CB/F307 3.8 (2.8) | CA/P309-OG/S300 4.3 (3.3)  CG/P309-O/V301 4.3 (3.4)  CG/P309-O/T302 3.3 (2.8)  CA/K308-O/D304 3.6 (2.6) |
| 41. 2,6-dihydropseudo-  oxynicotine hydrolase-like | 2JBW_A [43] | OE1/E301-CB/H329 4.4 (3.5)  CG2/V302-CE1/H329 5.3 | CG2/V302-O/G240 4.9 (3.9)  CG1/V302-O/G241 3.9 (2.8) | OD1/D300-N/V302 3.2  OD2/D300-CB/V302 4.0 (3.2) | CD2/L304-CE1/H296 3.6  O/V302-O/G297 4.8  O/V302-O/V298 4.8  O/V302-O/D300 3.1 |

**S1C Table. Structural characteristics of atoms forming weak hydrogen bonds in the conservative catalytic core of three ABH fold enzymes: acetylxylan esterase, hydroxynitrilase and haloalkane dehalogenase.** For acetylxylan esterase and hydroxynitrilase, structural characteristics are obtained on the basis of three-dimensional structures determined at cryogenic and room temperatures. For haloalkane dehalogenase, three structures were used: one structure determined at cryogenic temperature and two structures at room temperature, but with different pH values. All weak hydrogen bonds are divided into groups in accordance with the catalytic zones and publications in which they were studied. For each weak hydrogen bond, its geometric parameters in angstroms (Å) are given: the distance between the C and O atoms, as well as the distance between H and O (in brackets). In structures of the acetylxylan esterase (PDB ID:1G66_A, cryogenic temperature) and hydroxynitrilase (PDB ID:3C6X_A, cryogenic temperature) the coordinates of hydrogen atoms were determined experimentally and these coordinates were taken for calculations. In the remaining structures, the coordinates of the hydrogen atoms were calculated by the BIOVIA (Accelrys) Discovery Studio [45]. In addition, the value of the B-factor (Å^2^) is given for each atom of the weak hydrogen bonds. And again, for hydrogens in structures of the acetylxylan esterase (PDB ID:1G66_A, cryogenic temperature) and hydroxynitrilase (PDB ID:3C6X_A, cryogenic temperature), the experimental values of B-factors are given (in brackets). In other structures, the values of the B-factor of hydrogens (in brackets) are calculated by the BIOVIA (Accelrys) Discovery Studio. For only one carbon atom and its associated hydrogen atom, the values of the B-factor given in the table slightly exceed 20 Å^2^. In the structure of acetylxylan esterase (PDB ID:1G66_A, cryogenic temperature), there are two conformers for catalytic histidine 187.

| Acetylxylan esterase | |
| --- | --- |
| PDB ID:1G66_A, R=0.90 Å, Cryogenic temper. [46] | PDB ID:1BS9_A, R=1.10 Å, Room temperature [33] |
| Weak hydrogen bond (Å), B-factor (Å^2^) | Weak hydrogen bond (Å), B-factor (Å^2^) |
| Catalytic acid zone [47] | |
| CA/C171-OH/Y190 3.6 (2.8), 3.36 (4.03) - 4.01 | CA/C171-OH/Y190 3.7 (2.7), 7.35 (8.10) - 9.99 |
| O/D172-CB/D175 3.2 (2.7), 4.02 - 4.01 (4.82) | O/D172-CB/D175 3.2 (2.7), 8.56 - 8.39 (9.00) |
| OD1/D175-CA/G132 4.4 (3.6), 4.89 - 4.13 (4.96) | OD2/D175-CA/G132 4.6 (3.8), 9.57 - 7.98 (7.60) |
| CE1/H187-O/G132 3.1 (2.2), 7.85 (9.42) - 4.32  CE1/H187-O/G132 3.1 (2.2), 6.34 (7.60) - 4.32 | CE1/H187-O/G132 3.8 (3.2), 11.80 (12.42) - 7.98 |
| CA/G132-OH/Y190 4.9 (4.1), 4.13 (4.96) - 4.01 | CA/G132-OH/Y190 4.9 (4.1), 7.98 (7.60) - 9.99 |
| Nucleophile zone [48] | |
| O/Y89-CA/E12 3.4 (2.9), 4.02 - 4.13 (4.96) | O/Y89-CA/E12 3.4 (2.8), 8.42 - 8.23 (10.78) |
| CA/G92-O/R11 3.5 (3.0), 3.87 (4.65) - 4.37 | CA/G92-O/R11 3.6 (3.1), 8.31 (8.40) - 9.32 |
| CA/G88-O/A10 3.5 (2.7), 4.00 (4.80) - 3.89 | CA/G88-O/A10 3.5 (2.6), 7.80 (7.93) - 8.60 |
| CA/G88-O/G92 3.6 (2.8), 4.00 (4.80) - 4.17 | CA/G88-O/G92 3.7 (2.8), 7.80 (7.93) - 10.65 |
| CA/G92-O/A10 3.7 (2.7), 3.87 (4.65) - 3.89 | CA/G92-O/A10 3.8 (2.7), 8.31 (8.40) - 8.60 |
| Oxyanion zone [48] | |
| CG/R11-O/E12 3.6 (2.7), 4.01 (4.81) - 3.47 | CG/R11-O/E12 3.6 (2.6), 8.35 (9.10) - 8.96 |
| CA/A10-HOH403 3.6 (2.7), 4.02 (4.83) - 4.36 | CA/A10-HOH301 3.7 (2.7), 7.12 (8.13) - 9.85 |
| CG/R11-HOH403 3.2 (2.7), 4.01 (4.81) - 4.36 | CG/R11-HOH301 3.3 (2.6), 8.35 (9.10) - 9.85 |
| Catalytic triad^This work^ | |
| CA/S90-O/G132 3.4 (2.4), 3.97 (4.76) - 4.32 | CA/S90-O/G132 3.4 (2.3), 8.62 (10.30) - 8.57 |
| O/S90-CA/D133 4.0 (3.0), 3.75 - 3.67 (4.40) | O/S90-CA/D133 4.0 (2.9), 7.99 - 7.49 (8.12) |
| OE2/E94-CB/P134 3.5 (2.7), 6.29 - 5.08 (6.09) | OE1/E94-CG/P134 3.5 (2.8), 11.89 - 10.61 (8.12) |
| CA/H187-OD1/D175 3.3 (2.4), 5.56 (6.67) - 4.89  CA/H187-OD1/D175 3.2 (2.4), 5.47 (6.56) - 4.89 | CA/H187-OD2/D175 3.3 (2.4), 11.28 (12.42) - 9.57 |
| CB/H187-OD1/D175 3.6 (3.3), 7.11 (8.53) - 4.89  CB/H187-OD1/D175 3.3 (3.0), 5.26 (6.32) - 4.89 | CB/H187-OD2/D175 3.4 (3.1), 12.25 (12.42) - 9.57 |
| CB/C178-O/G132 3.7 (2.7), 5.11 (6.13) - 4.32 | CB/C178-O/G132 3.7 (2.6), 7.86 (8.02) - 8.57 |
| CB/C178-O/D133 3.1 (2.5), 5.11 (6.13) - 4.09 | CB/C178-O/D133 3.2 (2.5), 7.86 (8.02) - 7.62 |
| CB/C179-O/D172 3.3 (2.7), 3.54 (4.25) - 4.02 | CB/C179-O/D172 3.3 (2.7), 7.49 (7.96) - 8.56 |
| Hydroxynitrilase | |
| PDB ID:3C6X_A, R=1.05 Å, Cryogenic temp. [31] | PDB ID:6YAS_A, R=2.20 Å, Room temperature [49] |
| Weak hydrogen bond (Å), B-factor (Å^2^) | Weak hydrogen bond (Å), B-factor (Å^2^) |
| Catalytic acid zone [47] | |
| CA/W203-OD1/N104 3.2 (2.4), 8.38 (10.95) - 8.23 | CA/W203-OD1/N104 3.4 (2.5), 11.70 (12.67) - 14.77 |
| O/T204-CB/D207 3.4 (2.8), 9.42 - 10.24 (12.29) | O/T204-CB/D207 3.4 (2.7), 16.81 - 12.73 (13.65) |
| OD2/D207-CB/N104 3.3 (2.7), 9.58-8.05 (9.66) | OD2/D207-CB/N104 3.5 (2.8), 11.14 - 10.51 (11.64) |
| O/G233-CB/Q206 3.6 (3.0), 12.13 - 14.53 (17.44) | O/G233-CB/Q206 3.2 (2.6), 14.55-21.68 (25.07) |
| CE1/H235-O/N104 3.7 (3.0), 12.09 (14.51) - 8.63 | CE1/H235-O/N104 3.4 (2.6), 11.09 (12.54) - 11.06 |
| Nucleophile zone [48] | |
| O/E79-CB/T11 3.5 (2.7), 7.73 - 7.55 (9.06) | O/E79-CB/T11 3.6 (2.7), 12.96-11.48 (11.13) |
| CA/G82-O/H10 4.1 (3.7), 8.65 (10.38) - 6.95 | CA/G82-O/H10 4.0 (3.5), 8.82 (10.76) - 10.93 |
| CA/G78-O/I9 3.4 (2.6), 6.87 (8.24) - 6.27 | CA/G78-O/I9 3.4 (2.4), 10.52 (11.67) - 11.95 |
| CA/G78-O/G82 4.5 (3.7), 6.87 (8.24) - 8.03 | CA/G78-O/G82 4.5 (3.4), 10.52 (11.67) - 12.20 |
| CA/G82-O/I9 4.5 (3.6), 8.65 (10.38) - 6.27 | CA/G82-O/I9 4.6 (3.6), 8.82 (10.76) - 11.95 |
| Oxyanion zone [48] | |
| CD2/H10-O/L36 3.2 (2.4), 6.28 (7.53) - 10.47 | CD2/H10-O/T11 2.9 (1.8), 6.62 (8.45) - 10.42 |
| CA/I9-HOH2002 3.5 (2.9), 5.97 (7.17) - 7.67 | CA/I9-HOH524 3.7 (3.1), 8.51 (9.16) - 14.83 |
| CD2/H10-HOH2002 3.4 (2.6), 6.28 (7.53) - 7.67 | N/A |
| CA/A16-HOH2002 3.7 (2.8), 6.87 (8.24) - 7.67 | CA/A16-HOH524 3.6 (2.6), 7.79 (9.09) - 14.83 |
| Catalytic triad^This work^ | |
| CA/S80-O/N104 3.1 (2.4), 8.97 (10.77) - 8.63 | CA/S80-O/N104 3.2 (2.3), 11.48 (13.92) - 11.06 |
| O/S80-CA/S105, 3.2 (2.5), 10.81 - 7.93 (9.52) | O/S80-CA/S105, 3.2 (2.4), 14.00 - 11.63 (10.94) |
| CA/G83-O/V106 3.4 (2.5), 7.76 (9.31) - 8.10 | CA/G83-O/V106 3.6 (2.5), 9.78 (11.19) - 10.36 |
| CA/H235-OD2/D207 3.7 (2.9), 7.83 (9.39) - 9.58 | CA/H235-OD2/D207 3.8 (2.9), 11.71 (12.54) - 11.14 |
| CB/H235-OD1/D207 3.5 (2.7), 8.58 (10.29) - 9.62 | CB/H235-OD1/D207 3.4 (2.7), 13.06 (12.54) - 10.14 |
| CD2/F210-O/N104 3.3 (3.2), 11.42 (13.70) - 8.63 | CD2/F210-O/N104 3.1 (3.0), 12.71 (13.08) - 11.06 |
| CE2/F210-O/S105 4.5 (3.7), 12.38 (14.85) - 8.89 | CE2/F210-O/S105 4.4 (3.5), 13.30 (13.08) - 12.89 |
| OD2/D207-CB/F210 3.5 (2.5), 9.58 - 9.55 (11.46) | OD2/D207-CB/F210 3.4 (2.3), 11.14 - 12.30 (13.08) |
| OE1/Q215-CB/W203 3.2 (2.7), 8.84-9.15 (10.97) | OE1/Q215-CB/W203 3.3 (2.6), 12.19 - 11.77 (12.67) |
| CG/P212-O/D205 3.4 (2.6), 15.41 (18.49) - 13.79 | CG/P212-O/D205 3.4 (2.6), 10.34 (11.81) - 16.58 |
| CA/L211-O/D207 3.8 (2.9), 10.77 (12.93) - 11.70 | CA/L211-O/D207 3.8 (2.8), 13.33 (13.89) - 15.58 |

| Haloalkane dehalogenase | | |
| --- | --- | --- |
| PDB ID:1B6G_A, R=1.15 Å, pH 5.0  Cryogenic temperature [14] | PDB ID:2HAD_A^50^, R=1.90 Å, pH 6.2  Room temperature [50] | PDB ID:1EDE_A^50^, R=1.90 Å, pH 8.2  Room temperature [50] |
| Weak hydrogen bond (Å), B-factor (Å^2^) | Weak hydrogen bond (Å), B-factor (Å^2^) | Weak hydrogen bond (Å), B-factor (Å^2^) |
| Catalytic acid zone [47] | | |
| CA/I256-OD1/N148 3.4 ( 2.4), 10.01 (10.18) - 9.70 | CA/I256-OD1/N148 3.4 ( 2.5), 8.23 (8.35) - 9.92 | CA/I256-OD1/N148 3.5 ( 2.5), 9.25 (9.54) - 6.72 |
| O/G257-CB/D260 3.3 (2.9), 10.43 - 9.74 (10.25) | O/G257-CB/D260 3.3 (2.9), 7.67 - 7.83 (8.55) | O/G257-CB/D260 3.4 (2.9), 8.27 - 7.57 (7.72) |
| OD2/D260-CB/N148 3.6 (2.9), 9.92 - 9.74 (9.40) | OD2/D260-CB/N148 3.7 (2.9), 7.34 - 7.33 (7.86) | OD2/D260-CB/N148 3.7 (2.9), 6.18 - 5.65 (6.06) |
| CE1/H289-O/N148 4.7 (4.2), 14.05 (11.27) - 9.68 | CE1/H289-O/N148 4.5 (4.1), 7.69 (6.83) - 7.34 | CE1/H289-O/N148 4.8 (4.3), 6.95 (7.12) - 5.29 |
| Nucleophile zone [48] | | |
| O/Q123-CA/G55 4.3 (3.6), 9.25 - 9.70 (9.23) | O/Q123-CA/G55 4.2 (3.5), 6.20 - 6.18 (5.88) | O/Q123-CA/G55 4.2 (3.6), 6.53 - 5.67 (5.46) |
| CA/G126-O/H54 3.1 (2.8), 9.57 (9.52) - 9.59 | CA/G126-O/H54 3.0 (2.7), 5.64 (6.53) - 5.28 | CA/G126-O/H54 3.1 (2.9), 5.17 (5.64) - 3.98 |
| CA/V122-O/L53 4.1 (3.1), 9.80 (10.44) - 9.47 | CA/V122-O/L53 4.2 (3.1), 5.75 (5.97) - 4.29 | CA/V122-O/L53 4.2 (3.2), 5.68 (5.23) - 3.16 |
| CG1/V122-O/G126 3.4 (2.8), 12.94 (10.44) - 10.24 | CG1/V122-O/G126 3.5 (3.0), 6.28 (5.97) - 8.21 | CG1/V122-O/G126 3.4 (3.0), 3.63 (5.23) - 6.78 |
| CA/G126-O/L53 3.7 (2.6), 9.57 (9.52) - 9.47 | CA/G126-O/L53 3.9 (2.8), 5.64 (6.53) - 4.29 | CA/G126-O/L53 3.9 (2.9), 5.17 (5.64) - 3.16 |
| Oxyanion zone [48] | | |
| CD2/H54-O/P80 3.4 (2.4), 8.89 (9.03) - 9.94 | CD2/H54-O/P80 3.3 (2.4), 5.97 (4.80) - 7.06 | CD2/H54-O/P80 3.3 (2.3), 4.08 (3.76) - 7.25 |
| CA/L53-HOH2010 3.3 (2.4), 9.39 (9.91) - 10.42 | CA/L53-HOH409 3.5 (2.6), 5.34 (5.32) - 8.42 | CA/L53-HOH407 3.3 (2.3), 5.01 (5.25) - 7.68 |
| CD2/H54-HOH2010 3.3 (2.9), 8.89 (9.03) - 10.42 | CD2/H54-HOH409 3.2 (2.7), 5.97 (4.80) - 8.42 | CD2/H54-HOH407 3.3 (2.9), 4.08 (3.76) - 7.68 |
| CD2/H54-HOH2002 3.8 (2.8), 8.89 (9.03) - 10.72 | CD2/H54-HOH408 3.6 (2.7), 5.97 (4.80) - 8.09 | CD2/H54-HOH406 3.7 (2.7), 4.08 (3.76) - 6.43 |
| CA/S60-HOH2002 3.4 (2.6), 8.97 (9.20) - 10.72 | CA/S60-HOH408 3.5 (2.6), 3.63 (3.44) - 8.09 | CA/S60-HOH406 3.4 (2.6), 3.92 (3.17) - 6.43 |
| HOH2002-HOH2010 3.8, 10.72 - 10.42 | HOH408-HOH409 3.9, 8.09 - 8.42 | HOH406-HOH407 3.9, 6.43 - 7.68 |
| Catalytic triad^This work^ | | |
| O/D124-CB/A149 4.1 (3.5), 9.41 - 10.40 (10.19) | O/D124-CB/A149 4.2 (3.6), 3.68 - 5.46 (5.49) | O/D124-CB/A149 4.0 (3.5), 3.73 - 4.68 (5.33) |
| CA/F128-O/C150 3.6 (2.6), 10.01 (10.61) - 11.76 | CA/F128-O/C150 3.6 (2.7), 7.93 (7.72) - 7.97 | CA/F128-O/C150 3.6 (2.7), 7.65 (7.00) - 6.20 |
| CD1/F128-O/C150 3.3 (2.3), 10.66 (10.61) - 11.76 | CD1/F128-O/C150 3.2 (2.2), 6.83 (7.72) - 7.97 | CD1/F128-O/C150 3.2 (2.2), 6.86 (7.00) - 6.20 |
| CA/H289-OD2/D260 3.5 (2.6), 9.64 (11.27) - 9.92 | CA/H289-OD2/D260 3.6 (2.7), 6.54 (6.83) - 7.34 | CA/H289-OD2/D260 3.7 (2.7), 7.62 (7.12) - 6.18 |
| CB/H289-OD1/D260 3.2 (2.3), 9.66 (11.27) - 10.48 | CB/H289-OD1/D260 3.3 (2.4), 5.79 (6.83) - 7.11 | CB/H289-OD1/D260 3.5 (2.6), 6.82 (7.12) - 7.26 |
| CD1/L263-O/N148 3.7 (2.9), 14.05 (10.99) - 9.68 | CD1/L263-O/N148 3.5 (2.7), 11.39 (10.78) - 7.34 | CD1/L263-O/N148 3.5 (2.7), 8.66 (8.19) - 5.29 |
| OD2/D260-CB/L263 3.6 (2.8), 9.92 - 10.62 (10.99) | OD2/D260-CB/L263 3.8 (3.2), 7.34 - 10.21 (10.78) | OD2/D260-CB/L263 3.6 (3.0), 6.18 - 8.48 (8.19) |
| CA/G264-O/G257 4.4 (3.6), 10.88 (10.48) - 10.43 | CA/G264-O/G257 4.4 (3.5), 11.01 (10.57) - 7.67 | CA/G264-O/G257 4.3 (3.4), 8.41 (8.20) - 8.27 |
| CD/P265-O/M258 4.6 (4.0), 11.52 (12.71) - 12.87 | CD/P265-O/M258 4.5 (4.0), 13.08 (13.83) - 13.94 | CD/P265-O/M258 4.4 (3.9), 13.53 (13.53) - 11.89 |
| CA/G264-O/D260 4.0 (3.2), 10.88 (10.48) - 11.48 | CA/G264-O/D260 3.7 (3.0), 11.01 (10.57) - 9.92 | CA/G264-O/D260 3.7 (2.9), 8.41 (8.20) - 7.11 |

**References**

1. Ohara K, Unno H, Hosoya M, Fujino N, Hirooka K, Takahashi S, Yamasita S, et al. (2014) Structural insights into the low pH adaptation of a unique carboxylesterase from *Ferroplasma*: altering the pH optima of two carboxylesterases. J Biol Chem 289:24499-24510.

2. Murzin AG, Brenner SE, Hubbard T, Chothia C (1995) SCOP: a structural classification of proteins database for the investigation of sequences and structures. J Mol Biol 247:536-540.

3. Spiller B, Gershenson A, Arnold FH, Stevens RC (1999) A structural view of evolutionary divergence. Proc Natl Acad Sci U S A 96:12305-12310.

4. Zhu X, Larsen NA, Basran A, Bruce NC, Wilson IA (2003) Observation of an arsenic adduct in an acetyl esterase crystal structure. J Biol Chem 278:2008-2014.

5. Ronning DR, Klabunde T, Besra GS, Vissa VD, Belisle JT, Sacchettini JC (2000) Crystal structure of the secreted form of antigen 85C reveals potential targets for mycobacterial drugs and vaccines. Nat Struct Biol 7:141-146.

6. Murayama K, Shirouzu M, Terada T, Kuramitsu S, Yokoyama S (2005) Crystal structure of TT1662 from *Thermus thermophilus* HB8: a member of the alpha/beta hydrolase fold enzymes. Proteins 58:982-984.

7. Narasimhan D, Collins GT, Nance MR, Nichols J, Edwald E, Chan J, et al. (2011) Subunit stabilization and polyethylene glycolation of cocaine esterase improves in vivo residence time. Mol Pharmacol 80:1056-1065.

8. Szeltner Z, Rea D, Renner V, Fulop V, Polgar L (2002) Electrostatic effects and binding determinants in the catalysis of prolyl oligopeptidase. Site specific mutagenesis at the oxyanion binding site. J Biol Chem 277:42613-42622.

9. Engel M, Hoffmann T, Wagner L, Wermann M, Heiser U, Kiefersauer R, et al. (2003) The crystal structure of dipeptidyl peptidase IV (CD26) reveals its functional regulation and enzymatic mechanism. Proc Natl Acad Sci U S A 100:5063-5068.

10. Liao DI, Breddam K, Sweet RM, Bullock T, Remington SJ (1992) Refined atomic model of wheat serine carboxypeptidase II at 2.2Å resolution. Biochemistry 31:9796-9812.

11. Roussel A, Canaan S, Egloff MP, Rivière M, Dupuis L, Verger R, et al, (1999) Crystal structure of human gastric lipase and model of lysosomal acid lipase, two lipolytic enzymes of medical interest. J Biol Chem 274:16995-17002.

12. Goettig P, Groll M, Kim JS, Huber R, Brandstetter H (2002) Structures of the tricorn-interacting aminopeptidase F1 with different ligands explain its catalytic mechanism. EMBO J 21:5343-5352.

13. Zhang R, Koroleva O, Collert F, Joachimiak A, Midwest Center for Structural Genomics. 1.5 Å crystal structure of the cephalosporin C deacetylase.

14. Ridder IS, Rozeboom HJ, Dijkstra BW (1999) Haloalkane dehalogenase from Xanthobacter autotrophicus GJ10 refined at 1.15 A resolution. Acta Crystallogr D Biol Crystallogr 55:1273-1290.

15. Oakley AJ, Klvana M, Otyepka M, Nagata Y, Wilce MC, Damborsky J (2004) Crystal structure of haloalkane dehalogenase LinB from *Sphingomonas paucimobilis* UT26 at 0.95 A resolution: dynamics of catalytic residues. Biochemistry 43:870-878.

16. Kim HK, Liu JW, Carr PD, Ollis DL (2005) Following directed evolution with crystallography: structural changes observed in changing the substrate specificity of dienelactone hydrolase. Acta Crystallogr D Biol Crystallogr 61:920-931.

17. Horsman GP, Ke J, Dai S, Seah SY, Bolin JT, Eltis LD (2006) Kinetic and structural insight into the mechanism of BphD, a C-C bond hydrolase from the biphenyl degradation pathway. Biochemistry 45:11071-11086.

18. Agarwal V, Lin S, Lukk T, Nair SK, Cronan JE (2012) Structure of the enzyme-acyl carrier protein (ACP) substrate gatekeeper complex required for biotin synthesis. Proc Natl Acad Sci U S A 109:17406-17411.

19. Jansson A, Niemi J, Mantsala P, Schneider G (2003) Crystal structure of aclacinomycin methylesterase with bound product analogues: implications for anthracycline recognition and mechanism. J Biol Chem 278:39006-39013.

20. Seetharaman J, Lew S, Wang D, Kohan E, Patel D, Whitehead T, et al. Northeast Structural Genomics Consortium. Crystal Structure of Engineered Protein. Northeast Structural Genomics Consortium Target OR94.

21. Zou J, Hallberg BM, Bergfors T, Oesch F, Arand M, Mowbray SL, et al. (2000) Structure of *Aspergillus niger* epoxide hydrolase at 1.8 Å resolution: implications for the structure and function of the mammalian microsomal class of epoxide hydrolases. Structure 8:111-122.

22. Hofmann B, Tolzer S, Pelletier I, Altenbuchner J, van Pee KH, Hecht HJ (1998) Structural investigation of the cofactor-free chloroperoxidases. J Mol Biol 279:889-900.

23. Bellizzi III JJ, Widom J, Kemp C, Lu JY, Das AK, Hofmann SL, et al. (2000) The crystal structure of palmitoyl protein thioesterase 1 and the molecular basis of infantile neuronal ceroid lipofuscinosis. Proc Natl Acad Sci U S A 97:4573-4578.

24. Devedjiev Y, Dauter Z, Kuznetsov SR, Jones TL, Derewenda ZS (2000) Crystal structure of the human acyl protein thioesterase I from a single X-ray data set to 1.5 Å. Structure 8:1137-1146.

25. Padmanabhan B, Kuzuhara T, Adachi N, Horikoshi M (2004) The crystal structure of CCG1/TAF(II)250-interacting factor B (CIB). J Biol Chem 279:9615-9624.

26. Bourne PC, Isupov MN, Littlechild JA (2000) The atomic-resolution structure of a novel bacterial esterase. Structure 8:143-151.

27. Wei Y, Swenson L, Castro C, Derewenda U, Minor W, Arai H, et al. (1998) Structure of a microbial homologue of mammalian platelet-activating factor acetylhydrolases: *Streptomyces exfoliatus* lipase at 1.9 Å resolution. Structure 6:511-519.

28. Uppenberg J, Hansen MT, Patkar S, Jones TA (1994) The sequence, crystal structure determination and refinement of two crystal forms of lipase B from *Candida antarctica*. Structure 2:293-308.

29. Kawasaki K, Kondo H, Suzuki M, Ohgiya S, Tsuda S (2002) Alternate conformations observed in catalytic serine of *Bacillus subtilis* lipase determined at 1.3 Å resolution. Acta Crystallogr D Biol Crystallogr 58:1168-1174.

30. Roussel A, Yang Y, Ferrato F, Verger R, Cambillau C, Lowe M (1998) Structure and activity of rat pancreatic lipase-related protein 2. J Biol Chem 273:32121-32128.

31. Schmidt A, Gruber K, Kratky C, Lamzin VS (2008) Atomic resolution crystal structures and quantum chemistry meet to reveal subtleties of hydroxynitrile lyase catalysis. J Biol Chem 283:21827-21836.

32. Bruner SD, Weber T, Kohli RM, Schwarzer D, Marahiel MA, Walsh CT, et al. (2002) Structural basis for the cyclization of the lipopeptide antibiotic surfactin by the thioesterase domain SrfTE. Structure 10:301-310.

33. Ghosh D, Erman M, Sawicki M, Lala P, Weeks DR, Li N, et al. (1999) Determination of a protein structure by iodination: the structure of iodinated acetylxylan esterase. Acta Crystallogr D Biol Crystallogr 55:779-784.

34. Janda IK, Devedjiev Y, Cooper DR, Chruszcz M, Derewenda U, Gabrys A, et al. (2004) Harvesting the high-hanging fruit: the structure of the YdeN gene product from *Bacillus subtilis* at 1.8 angstroms resolution. Acta Crystallogr D Biol Crystallogr 60:1101-1107.

35. Arndt JW, Schwarzenbacher R, Page R, Abdubek P, Ambing E, Biorac T, et al. (2005) Crystal structure of an alpha/beta serine hydrolase (YDR428C) from *Saccharomyces cerevisiae* at 1.85 Å resolution. Proteins 58:755-758.

36. Bartlam M, Wang G, Yang H, Gao R, Zhao X, Xie G, et al. (2004) Crystal structure of an acylpeptide hydrolase/esterase from *Aeropyrum pernix* K1. Structure 12:1481-1488.

37. Legler PM, Kumaran D, Swaminathan S, Studier FW, Millard CB (2008) Structural characterization and reversal of the natural organophosphate resistance of a D-type esterase, *Saccharomyces cerevisiae* S-formyl-glutathione hydrolase. Biochemistry 47:9592-9601.

38. Gorman J, Shapiro L, Burley SK, Midwest Center for Structural Genomics. Structural Genomics target NYSGRCT920 related to A/B-hydrolase fold.

39. Osipiuk J, Xu X, Zheng H, Savchenko A, Edwards A, Joachimiak A, Midwest Center for Structural Genomics. Crystal structure of hypothetical protein Atu1826, a putative alpha/beta hydrolase from *Agrobacterium tumefaciens*.

40. Hisano T, Kasuya K, Tezuka Y, Ishii N, Kobayashi T, Shiraki M, et al. (2006) The crystal structure of polyhydroxybutyrate depolymerase from *Penicillium funiculosum* provides insights into the recognition and degradation of biopolyesters. J Mol Biol 356:993-1004.

41. Larsen NA, Lin H, Wei R, Fischbach MA, Walsh CT (2006) Structural characterization of enterobactin hydrolase IroE. Biochemistry 45:10184-10190.

42. Mirza IA, Nazi I, Korczynska M, Wright GD, Berghuis AM (2005) Crystal structure of homoserine transacetylase from *Haemophilus influenzae* reveals a new family of alpha/beta-hydrolases. Biochemistry 44:15768-15773.

43. Schleberger C, Sachelaru P, Brandsch R, Schulz GE (2007) Structure and action of a C-C bond cleaving alpha/beta-hydrolase involved in nicotine degradation. J Mol Biol 367:409-418.

44. Wan WY, Milner-White EJ (1999) A natural grouping of motifs with an aspartate or asparagine residue forming two hydrogen bonds to residues ahead in sequence: their occurrence at alpha-helical N termini and in other situations. J Mol Biol 286:1633-1649.

45. Discovery Studio Modeling Environment, Release 2017, San Diego: Dassault Systemes; 2016.

46. Ghosh D, Sawicki M, Lala P, Erman M, Pangborn W, Eyzaguirre J, et al. (2001) Multiple conformations of catalytic serine and histidine in acetylxylan esterase at 0.90 Å. J Biol Chem 276:11159-11166.

47. Dimitriou PS, Denesyuk AI, Takahashi S, Yamashita S, Johnson MS, Nakayama T, et al. (2017) Alpha/beta-hydrolases: A unique structural motif coordinates catalytic acid residue in 40 protein fold families. Proteins 85:1845-1855.

48. Dimitriou PS, Denesyuk AI, Nakayama T, Johnson MS, Denessiouk K (2019) Distinctive structural motifs co-ordinate the catalytic nucleophile and the residues of the oxyanion hole in the alpha/beta-hydrolase fold enzymes. Protein Sci 28:344-364.

49. Zuegg J, Gruber K, Gugganig M, Wagner UG, Kratky C (1999) Three-dimensional structures of enzyme-substrate complexes of the hydroxynitrile lyase from *Hevea* *brasiliensis*. Protein Sci 8:1990-2000.

50. Franken SM, Rozeboom HJ, Kalk KH, Dijkstra BW (1991) Crystal structure of haloalkane dehalogenase: an enzyme to detoxify halogenated alkanes. EMBO J 10:1297-1302.
